# Culture and multiomic analysis of lung cancer patient-derived pleural effusions revealed distinct druggable molecular types

**DOI:** 10.1038/s41598-022-10318-5

**Published:** 2022-04-15

**Authors:** Ha-Young Seo, Soon-Chan Kim, Woo-lee Roh, Young-Kyoung Shin, Soyeon Kim, Dong-Wan Kim, Tae Min Kim, Ja-Lok Ku

**Affiliations:** 1 Korean Cell Line Bank, Laboratory of Cell Biology, Cancer Research Institute, Seoul National University College of Medicine, 103, Daehak-ro, Jongno-gu, Seoul, 03080 Korea; 2 Department of Biomedical Sciences, Seoul National University College of Medicine, Seoul, 03080 Korea; 3 Cancer Research Institute, Seoul National University, Seoul, 03080 Korea; 4 Ischemic/Hypoxic Disease Institute, Seoul National University College of Medicine, Seoul, 03080 South Korea; 5 Department of Internal Medicine, Seoul National University Hospital, Seoul, 03080 Korea

**Keywords:** Non-small-cell lung cancer, Cancer genomics, Diagnostic markers

## Abstract

Malignant pleural effusion (MPE) is an independent determinant of poor prognostic factor of non-small cell lung cancer (NSCLC). The course of anchorage independent growth within the pleural cavity likely reforms the innate molecular characteristics of malignant cells, which largely accounts for resistance to chemotherapy and poor prognosis after the surgical resection. Nevertheless, the genetic and transcriptomic features with respect to various drug responses of MPE-complicated NSCLC remain poorly understood. To obtain a clearer overview of the MPE-complicated NSCLC, we established 28 MPE-derived lung cancer cell lines which were subjected to genomic, transcriptomic and pharmacological analysis. Our results demonstrated MPE-derived NSCLC cell lines recapitulated representative driver mutations generally found in the primary NSCLC. It also exhibited the presence of distinct translational subtypes in accordance with the mutational profiles. The drug responses of several targeted chemotherapies accords with both genomic and transcriptomic characteristics of MPE-derived NSCLC cell lines. Our data also suggest that the impending drawback of mutation-based clinical diagnosis in evaluating MPE-complicated NSCLS patient responses. As a potential solution, our work showed the importance of comprehending transcriptomic characteristics in order to defy potential drug resistance caused by MPE.

## Introduction

Lung cancer is one of the most prevalent cause of cancer-related death worldwide, with an estimated one million deaths annually. Within Korean population, it is the most lethal disease and the third common types of cancer^1,2^. It is also the most prevailing cause of malignant pleural effusion (MPE), accounting for approximately 30% of such effusions^3^. Although recent advances in prevention and early detection have contributed to the general increase in the survival rate, the occurrence of MPE in the context of lung cancer designates terminal stage and an independent prognostic parameter of the poorer survival rate^4^. Lung cancer patients with MPE mostly exhibit insensitivities to certain chemotherapies mainly due to high tumor burden^5,6^. Besides, the course of acquiring anoikis resistance alters multiple tumorigenic pathways, which eventually brings about insensitivity to certain chemotherapies^7^. Nevertheless, the genetic and transcriptomic features with respect to various drug responses of MPE-complicated NSCLC have not been thoroughly studied yet, which makes the implication of drug insensitivities of MPE-derived tumor cell still hypothetical.

Moreover, several studies have indicated that acquired resistance to second line chemotherapy such as crizotinib is associated with aggressive disease progressions^8^. Nevertheless, the widely accepted oncogenic association of secondary or tertiary alternations by the second line chemotherapy has been focused on the primary NSCLC, and oncogenic impact of chemotherapy-derived molecular changes in MPE remains unclear.

To obtain a clearer overview of the MPE-complicated NSCLC in accordance with heterogeneous drug responses, we established 28 lung cancer cell lines derived directly from the MPE, and two crizotinib-resistant sublines which were then subjected to genomic, transcriptomic and pharmacological analysis. Our results not only showed that MPE-derived NSCLC cell lines recapitulated representative genetic aberrations of the primary NSCLC including epidermal growth factor receptor (*EGFR*) and anaplastic lymphoma kinase (*ALK*) fusion with other domains, but also demonstrated the presence of distinct translational subtypes in accordance with the mutational profiles, which was consistently reflected to the drug responses of certain targeted chemotherapies. Our data also suggest that the imminent downside of mutation-based clinical diagnosis in assessing MPE-complicated NSCLS patient responses. As a potential solution, our data showed the importance of comprehending transcriptomic characteristics in order to defy potential drug resistance caused by MPE.

## Results

### MPE-derived human lung adenocarcinoma cell lines displayed various growth patterns

**Figure 1:**
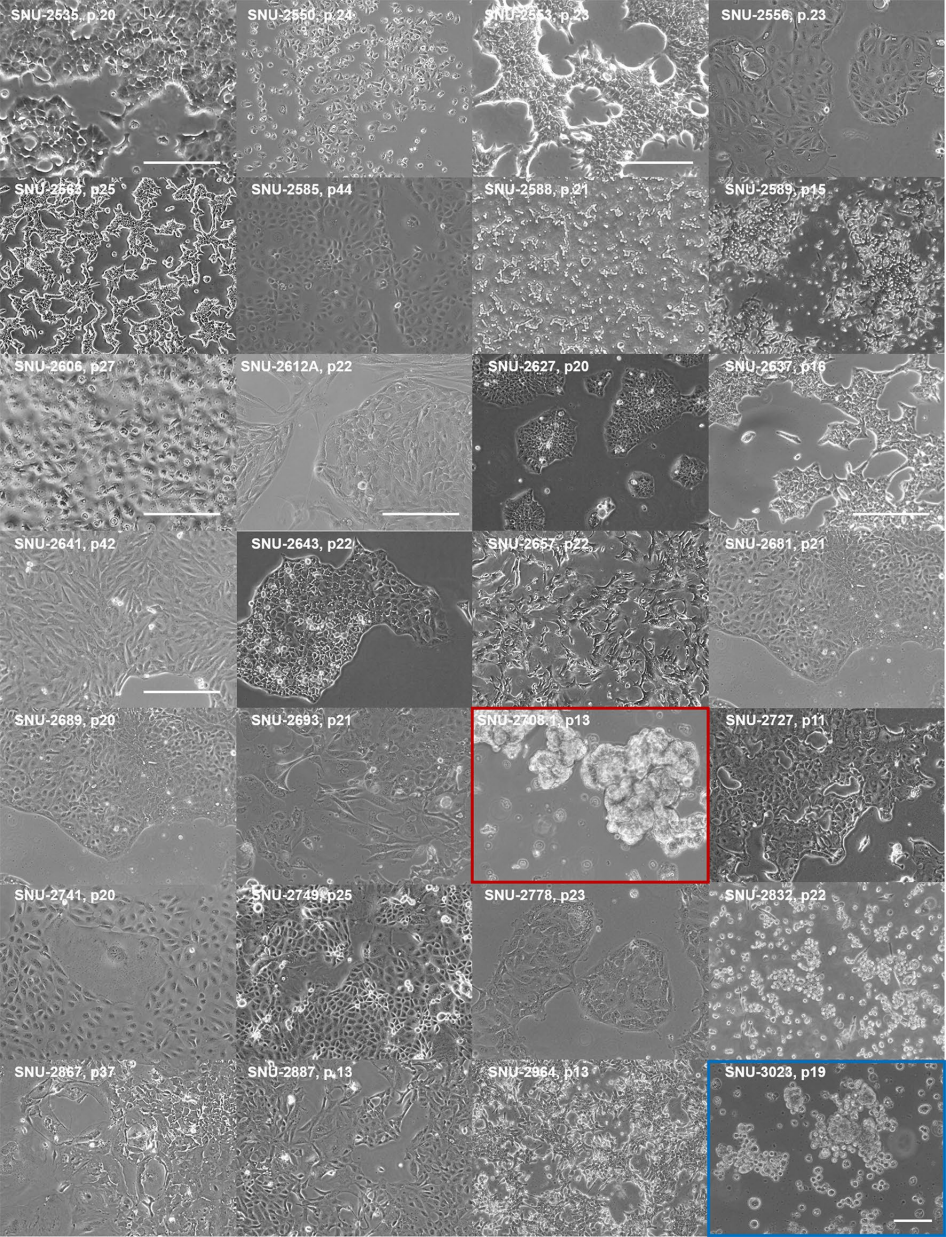
The morphologies of MPE-derived human lung adenocarcinoma cell lines exhibited heterogeneous growth patterns. Most cell lines grow as adherent form, suggesting cells floating in the pleural cavity maintained the capability to adhere. Various growth patterns include polygonal (*n* = 14), fibroblast-like (*n* = 3), round (*n* = 2), oval (*n* = 5) and mixed (*n* = 4) form. Scale bar = 100 μM.

The morphologies of the established cell lines were mainly categorized into four subtypes: polygonal, oval, fibroblast-like, and round (Fig. 1, Table 1). The doubling time of each cell lines varied with a range of 25 to 102 h (Table 1). We made sure the confluency of each cell lines are 50–80% so that the morphologies of cell lines were not affected by different level of contact inhibition. Although original tumor cells were derived from the MPE, which was expected to have resistance to anoikis, only two cell lines (SNU-2708.1 and SNU-3023) formed floating aggregates exhibiting round morphology. This suggests that all but two floating cell lines retained the nature of adherent growth patterns in the pleural cavity. There was also intra-morphological heterogeneity within a same cell population. For instance, the culture of SNU-2681 exhibited both round and polygonal tumor cells, which indicates the heterogeneous lung adenocarcinoma populations were retained in the in vitro culture.

**Table 1:** Clinicopathological features of 28 human lung cancer cell lines.

Cell Line	Culture site	Sex	Age	Pathology	Growth pattern	Doubling time(hr)	Cell morphology	Driver Event(s)
SNU-2535	Pleural effusion	F	56	ADC	Adherent	61	Polygonal	EML4(exon13)-ALK(exon20) fusion, FGFR4 (G388R)
SNU-2550	Pleural effusion	F	35	ADC	Adherent	32	Oval	EML4(exon6)-ALK(exon20) fusion
SNU-2553	Pleural effusion	M	40	ADC	Adherent	61	Fibroblast-like	EML4(exon20)-ALK(exon20) fusion
SNU-2556	Pleural effusion	F	72	ADC	Adherent	35	Oval	EGFR (E746_A750del), FGFR4 (G388R), TP53 (G154V)
SNU-2563	Pleural effusion	M	49	ADC	Adherent	47	Polygonal	EML4(exon18)-ALK(exon20) fusion, TP53 (R280K)
SNU-2585	Pleural effusion	M	75	ADC	Adherent	31	Oval	KRAS (G12V)
SNU-2588	Pleural effusion	M	43	ADC	Adherent	39	Polygonal	FGFR4 (G388R), TP53(P72R, G266R)
SNU-2589	Pleural effusion	F	52	ADC	Adherent	33	Polygonal	ERBB2 (I655V), TP53 (W146*)
SNU-2606	Pleural effusion	F	51	ADC	Adherent	28	Oval	CD74(exon6)-ROS1(exon34) fusion, FGFR4 (G388R), TP53 (R280K)
SNU-2612A	Pleural effusion	M	33	ADC	Adherent	39	Fibroblast-like	KIF5B(exon16)-RET(exon12) fusion
SNU-2627	Pleural effusion	F	46	NOS	Adherent	40	Polygonal	FGFR4 (G388R)
SNU-2637	Pleural effusion	M	42	ADC	Adherent	30	Polygonal	EML4(exon18)-ALK(exon20) fusion
SNU-2641	Pleural effusion	M	64	NOS	Adherent	44	Oval	TP53 (S183*)
SNU-2643	Pleural effusion	F	57	NOS	Adherent	27	Polygonal	EGFR (E746_A750del), FGFR4 (G388R), TP53 (R273C)
SNU-2657	Pleural effusion	F	70	ADC	Adherent	27	Polygonal	BRAF (V600E), ERRB2 (I600V), FGFR4 (G388R)
SNU-2681	Pleural effusion	M	63	ADC	Adherent	34	Round/Polygonal	ERBB2 (I655V), KRAS (G12C), TP53 (R273C)
SNU-2689	Pleural effusion	F	74	ADC	Adherent	25	Fibroblast-like/Oval	EGFR (L858R), ERBB2 (I655V), FGFR4 (G388R), TP53(I195T)
SNU-2693	Pleural effusion	M	60	ADC	Adherent	34	Polygonal/Oval	TP53 (P151H)
SNU-2708.1	Pleural effusion	M	56	ADC	Floating	–	Round	EGFR exon19 deletion (L747_T751del), TP53 (G245V)
SNU-2727	Pleural effusion	F	39	ADC	Adherent	102	Polygonal	EGFR (L858R), ERBB2 (I655V), FGFR4 (G388R), TP53 (S166*)
SNU-2741	Pleural effusion	M	57	ADC	Adherent	55	Polygonal	FGFR4 (G388R), EGFR (L747_P753delinsS), TP53 (P72R, V143M)
SNU-2749	Pleural effusion	M	67	ADC	Adherent	31	Polygonal	BRAF (V600E), ERBB2 (I655V), TP53 (Q331*), FGFR4 (G388R)
SNU-2778	Pleural effusion	M	54	ADC	Adherent	35	Polygonal	KIF5B(exon15)-RET(exon12) fusion, FGFR4 (G388R)
SNU-2832	Pleural effusion	F	58	ADC	Adherent	25	Fibroblast-like	CD74(exon6)-ROS1(exon34) fusion, ERBB2 (I655V)
SNU-2867	Pleural effusion	M	57	ADC	Adherent	87	Fibroblast-like/Polygonal	EGFR (E746fs, L747fs), TP53 (H179R)
SNU-2887	Pleural effusion	F	56	ADC	Adherent	102	Polygonal	EGFR (E746_A750del)
SNU-2964	Pleural effusion	F	78	ADC	Adherent	46	Polygonal	EGFR (E746_A750del)
SNU-3023	Pleural effusion	F	70	ADC	Floating	29	Round	EGFR (L858R), FGFR4 (G388R)

*ADC* indicates adenocarcinoma, *NOS* indicates not otherwise specified.

DNA fingerprinting analysis identified a heterogeneous distribution of 15 tetranucleotide repeat loci and an Amelogenin gender determining marker in each cell line, and confirmed 28 unique cell lines without cross-contamination (Supplementary Table 1). Although each cell lines were confirmed to be not cross-contaminated, the STR authentication between the cells harvested from MPE and matched cell lines has not been performed, which partially limited the association of clinical information. All cell lines were confirmed to be free of contamination from mycoplasma (Supplementary Fig. 1). The clinicopathological information are summarized in Table 1.

### MPE-derived lung cancer cell lines retain the representative genetic mutations of human lung adenocarcinoma

**Figure 2:**
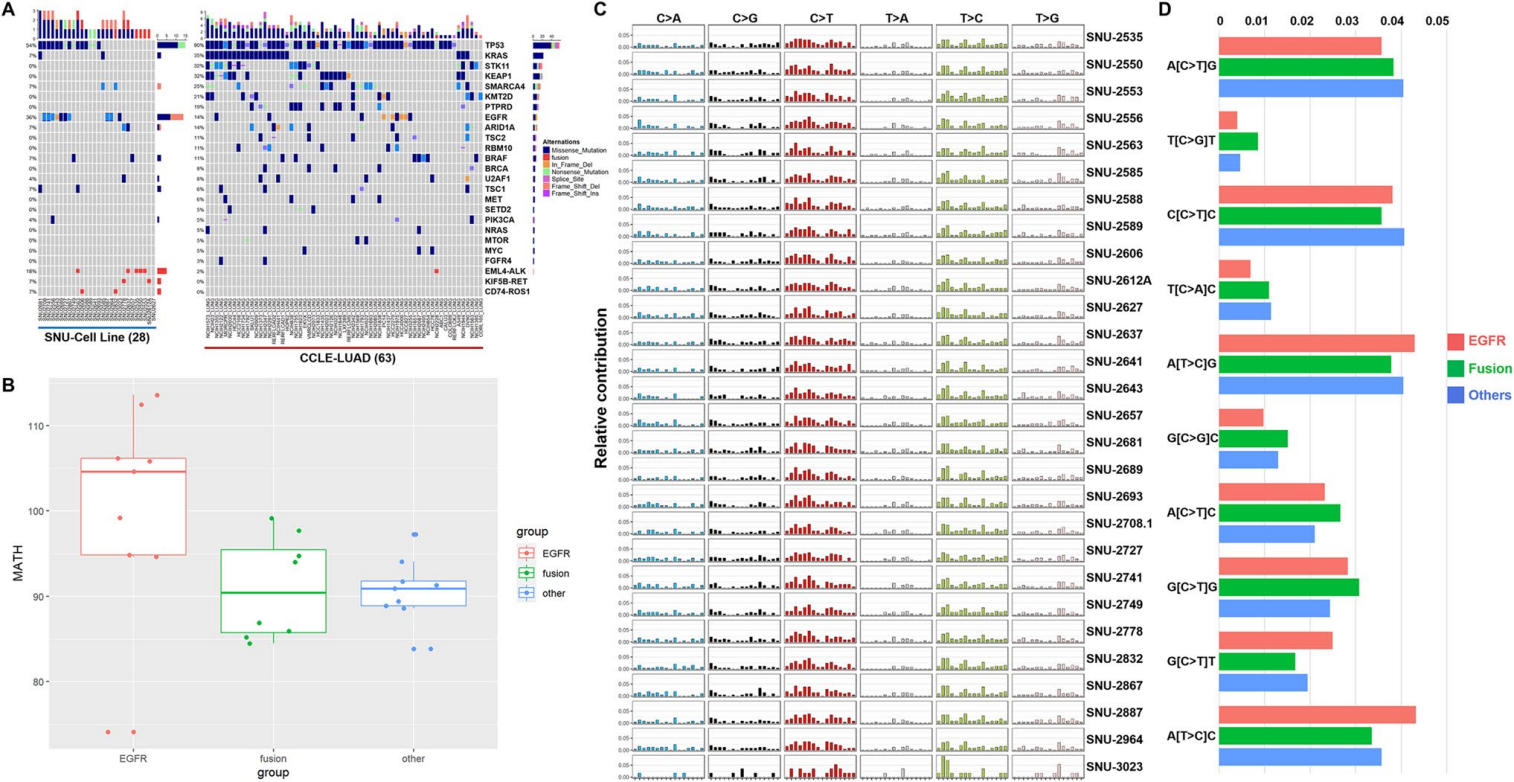
The characteristic genetic mutations of MPE-derived lung cancer cell lines. (**A**) See also Table 1. Comparison between the mutational landscape of MPE-derived cell lines (*n* = 28) and CCLE-LUAD cell lines (*n* = 63). MPE-derived cell lines harbored higher rates of the *EGFR* mutation as well as the fusion events. Each type of mutations is marked with representative colors on the waterfall plot. The total mutational loads are indicated on the top (per each cell lines) and right side (per each cohorts) of the plot. The percentage of mutational occurrence within the cohort is specified on the left side of the plot. (**B)** See also Supplementary Table 3. The MATH score demonstrated *EGFR*-mutant group had higher genetic heterogeneity score. Interquartile ranges with the median value are indicated on the box plot. Each group is marked with representative colors. (**C)** The relative contribution of SNVs for each cell lines. Most cell lines exhibited similar patterns except for the SNU-3023. Six different substitution types are designated with representative colors. (**D)** Top 10 different SNV contribution between the mutational subtypes. Each groups are marked with representative colors.

Several studies have shown that MPE-derived tumor cell lines maintain the mutational profiles of the lung adenocarcinoma^6,9^. We performed both whole exome sequencing (WES) and targeted sequencing (CCP) on the 28 MPE-derived cell lines. The overall mutational spectrum of the MPE-derived NSCLC cell lines such as mutant-allele tumor heterogeneity (MATH) analysis and relative contribution of mutational signatures was analyzed using WES data. The targeted sequencing as well as direct Sanger sequencing re-confirmed the presence of key driver mutations minimizing the possible false-positive sequencing errors (Supplementary Fig. 2, Supplementary Table 2). The precise exclusion of the germinant mutations was not feasible since the DNA from matched blood or normal tissue was unavailable when WES of established cell lines was performed. Alternatively, we have referred to the Clinvar database (https://www.ncbi.nlm.nih.gov/clinvar) in order to avoid misestimating the mutation frequency due to inflated germinant/benign mutations. The pathogenicity of the representative driver mutations was manually inspected, and all marked mutations for calculating the frequency of mutated tumor genes in Fig. 2A were previously reported as “Pathogenic”, “Likely Pathogenic” or “Drug Response” in the Clinvar database.

To establish a more comprehensive mutational profile, the aberrations in key driver genes were compared to a larger lung cancer cell line cohort from the cancer cell line encyclopedia project (CCLE)^10^ which was composed of a total of 63 lung cancer cell lines (Fig. 2A). As a first validation, our cohort had a significantly higher rate of *EGFR* mutations (36%) compared to CCLE cohort (14%). It has been reported that the *EGFR* mutation frequency in lung adenocarcinoma is approximately 10% as much larger cohort (TCGA)^11^ indicated (Supplementary Fig. 3). Pervasive *EGFR* mutation in our cohort was accordant with the previous MPE of lung adenocarcinoma study^12^. In contrast, *KRAS* mutation was detected at a low rate in our cohort (7%) compared to CCLE (35%) and TCGA (30%) cohorts, which was unexpected since mutant *KRAS* is reported to promote malignant pleural effusion formation^5^. Previous study indicated that *EGFR* is more frequently aberrated than *KRAS* in Korean NSCLC population^13^, which suggest that the geographic factor partially shaped the mutational landscape of our cohort. Nine patients harbored driver fusion genes including *EML4*-*ALK*, *KIF5B*-*RET* and *CD74*-*ROS1* in our cohort (Supplementary Fig. 4). Few groups reported that lung cancer patients with fusion genes, especially *EML4*-*ALK* were younger than those without^14,15^, which was in parallel with our data. The presence of fusion genes was mutually exclusive with *EGFR* and *KRAS* mutation in parallel with prior study^14^.

We also calculated the heterogeneity scores by using a previously reported method, mutant-allele tumor heterogeneity (MATH)^16^. This analysis allowed to estimate the dispersion of mutant allele frequencies (MAFs) within a cell population, which reflected the intra-tumor genetic heterogeneity. The cell lines with mutant *EGFR* had higher MATH score than two other groups, suggesting that higher level of genetic heterogeneity is causative factor for *EGFR* mutation (Fig. 2B). Then, we evaluated the relative contribution of single nucleotide variations (SNVs) to the mutational signature of each cell line using web-based algorithm, MUSICA. The most predominant point mutation type in the cell lines was the C-to-T transitions (Fig. 2C, Supplementary Table 3). Except for SNU-3023 that exhibited distinct configurations of the relative contribution of SNVs, all cell lines had analogous patterns. We then compared the most various mutational contribution among the three groups. A[T > C]C was the most different SNV signature followed by G[C > T]T (Fig. 2D).

Overall, comprehensive mutational analysis indicated that there is a distinct separation of genomic drivers in our cohort: *EGFR* aberration, fusion genes, and others. We have applied these groups for further analysis of transcriptome and drug screening.

### Transcriptomic analysis of MPE-derived lung cancer cell lines reveal a separation of EGFR and fusion types

**Figure 3:**
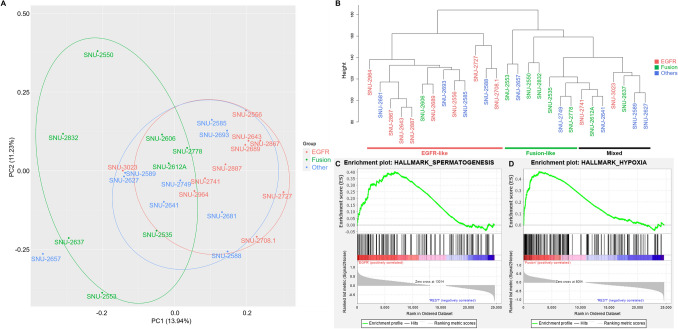
EGFR and fusion subtypes are separated in transcriptomic patterns. (**A**) Principle component analysis indicated that MPE-derived cell lines with fusion events exhibited higher transcriptional heterogeneity than *EGFR*-mutant types. The principle component 1 and 2 accounted for 13.94% and 11.23% of the total loading components respectively. Each group is marked with representative colors. (**B)** Hierarchal clustering analysis demonstrated there are characteristic expressional patterns between *EGFR*-mutant and fusion gene groups. Cell lines with mutated genes other than the *EGFR* and fusion gene are not clustered. Each groups are marked with representative colors. (**C)** See also Supplementary Table 4. GSEA analysis revealed that spermatogenesis pathway is upregulated in EGFR-mutant groups compared to the other cell lines. (**D)** See also Supplementary Table 4. Hypoxia pathway is distinctively expressed in fusion gene group. The statistical settings for GSEA analysis is as follows (Number of permutations = 1000, Permutation type = phenotype, Chip platform = MSigDB.v.7.4.chip, Enrichment statistic = weighted, Max size: exclude larger sets = 500, Min size: exclude smaller sets = 15).

We then verified that the pattern of mRNA expression reflects the distinctive genomic aberrations. The principle component analysis (PCA) of the mRNA expression indicated that MPE-derived cell lines were likely grouped in accordance with their genetic factors (Fig. 3A). Cell lines with *EGFR* mutation were specifically clustered with less degree of dispersion on the PCA plot compared to the fusion group, which suggests that *EGFR*-mutated NSCLCs in MPE display characteristic transcription patterns. Hierarchical clustering analysis also displayed that cell lines with the fusion genes were clustered distinctly from the cell lines with *EGFR* mutation (Fig. 3B). The other group consisting of cell lines with genomic aberrations other than *EGFR* and fusion genes were not clustered at all. Next, we performed gene set enrichment assay (GSEA) to identify pathways that are specifically enriched in each group. Only spermatogenesis pathway was significantly enriched in EGFR group compared to other two groups (*p* < 0.05). Multiple tumor driver genes that are associated with spermatogenesis pathway such as *mTOR*, *EZH2*, *NF2*, *DCC* and *MLF1* had high enrichment score in the EGFR group (Fig. 3C). We further validated the enriched-pathways in EGFR-mutant cell lines using CCLE dataset. We performed gene set enrichment analysis (GSEA) using the CCLE dataset restricted to EGFR mutated NSCLC cell lines that were introduced in mutational analysis section (Fig. 2A). The EGFR-mutant cell lines from the CCLE dataset were HCC2279, HCC4006, HCC827, NCIH1355, NCIH1793, NCIH1975, NCIH2291, NCIH3255 and PC14. We unified all running parameters of GSEA and the input matrix by accounting only shared genes between CCLE and our mRNA expression dataset which consists of 15,970 genes. Then, the gene sets that are upregulated in EGFR-mutated cell lines from CCLE were estimated in comparison with cell lines without EGFR mutation from our cohort. Indeed, spermatogenesis pathway was highly enriched at nominal *p*-value < 0.005. In contrast to our cohort, the GSEA using CCLE dataset identified G2M_checkpoint and E2F_targets pathways as significantly enriched at nominal *p*-value < 0.01 as well. This analysis reiterates the EGFR-mutated NSCLC indeed has distinctive transcriptomic subtype represented by spermatogenesis pathway in GSEA. Meanwhile, several pathways were enriched in the fusion gene group (Fig. 3D): Inflammatory response (*p* < 0.01), Hypoxia (*p* < 0.01), TNFα signaling (*p* < 0.05), Angiogenesis (*p* < 0.05). Genes with high enrichment scores for each signaling pathway are summarized in Supplementary Table 4. GSEA analysis designates that the transcriptomic aberrations in the fusion gene group exhibited more specific patterns in tumor-related pathways than the EGFR group.

### Induced resistance to crizotinib causes nuclear localization of β-catenin in MPE-derived NSCLC through increased DKK1 expression

**Figure 4:**
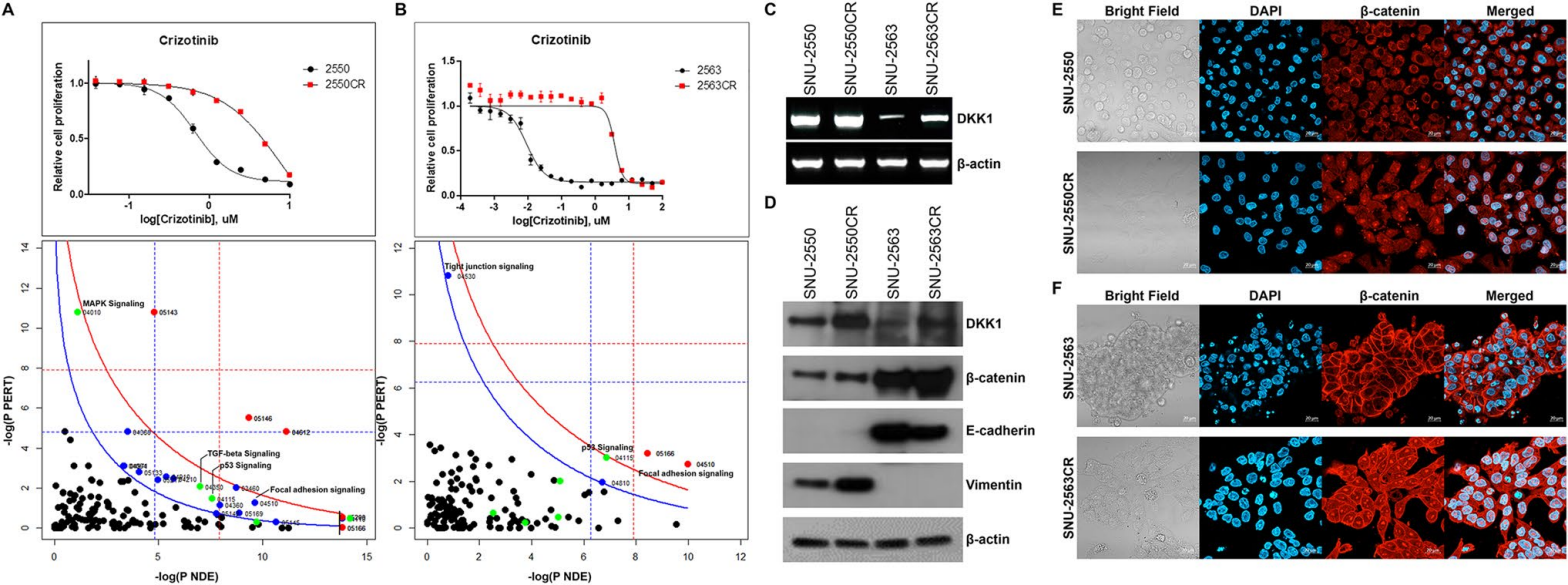
Repetitive exposure to crizotinib causes nuclear localization of β-catenin in MPE-derived NSCLC through increased DKK1 expression. (**A**,**B**) Two cell lines with *EML4-ALK* fusion were repetitively exposed to crizotinib in order to induce crizotinib resistance (CR). Both cell lines exhibited poor responses compared to their parental cell lines. Within the SPIA evidence plot, a single point represents each pathway and the coordinates are the log of over-representation *p*-value (pNDE) and the *p*-value from perturbations (pPERT). The oblique lines in the plot show the significance regions based on the combined evidence. The pathways at the right of the red curve are significant after Bonferroni correction of the global *p*-values, obtained by combining the pPERT and pNDE using the normal inversion method. The pathways at the right of the blue curve line are significant after a FDR correction of the global *p*-values. (**C)** The mRNA level of *DKK1* was increased on both CR sublines. (**D)** The protein level of *DKK1* was augmented as well on both CR sublines. The increased DKK1 barely affected the expression of β-catenin and E-cadherin. (**E,F)** Distinct nuclear localization of β-catenin was observed by both CR sublines. Scale bar = 20 μM.

An ALK inhibitor is the standard treatment for advanced NSCLC patients harboring the anaplastic lymphoma kinase (ALK) fusion gene. However, secondary *ALK* mutations or alternative pathway changes cause a fraction of the tumors to be resistant to ALK inhibitors^17,18^. We further validated that the acquired resistance to crizotinib, the first generation of the ALK inhibitor, alters the transcriptional patterns of MPE-derived NSCLC cell lines harboring EML4-ALK fusion gene. We established two crizotinib-resistant sublines (SNU-2550CR and SNU-2563CR) by long-term exposure to increasing concentrations of crizotinib, and performed RNA-seq. Both sublines exhibited distinct insensitivities to crizotinib, and a list of differentially expressed genes and their log fold changes together with signaling pathways topology were identified in order to detect the pathways most relevant to the crizotinib resistance. SPIA two-way analysis of RNA-seq revealed multiple signaling aberrations including p53 signaling and focal adhesion (Fig. 4A,B). Specifically, the mRNA level of *DKK1* which has been reported to promote the migration and invasion of NSCLC by inhibiting the phosphorylation of β-catenin^19,20^ was significantly upregulated in both crizotinib resistant cell lines. The elevated expression of *DKK1* was confirmed in mRNA (Fig. 4C) and protein (Fig. 4D) level. We further investigated the potential role of increased DKK1 expression in the context of β-catenin signaling. Immunocytochemistry indicated that the nuclear localization of β-catenin was significantly increased in both crizotinib-resistant cell lines (Fig. 4E,F).

### MPE-derived NSCLC cell lines reveal heterogeneous drug responses caused by molecular diversity

We assembled an 18-compound library for drug screening. In total, 30 cell lines including two crizotinib resistant sublines from 28 patients were successfully screened in experimental triplicate, generating > 500 measurements of cell line-drug interactions.

**Figure 5:**
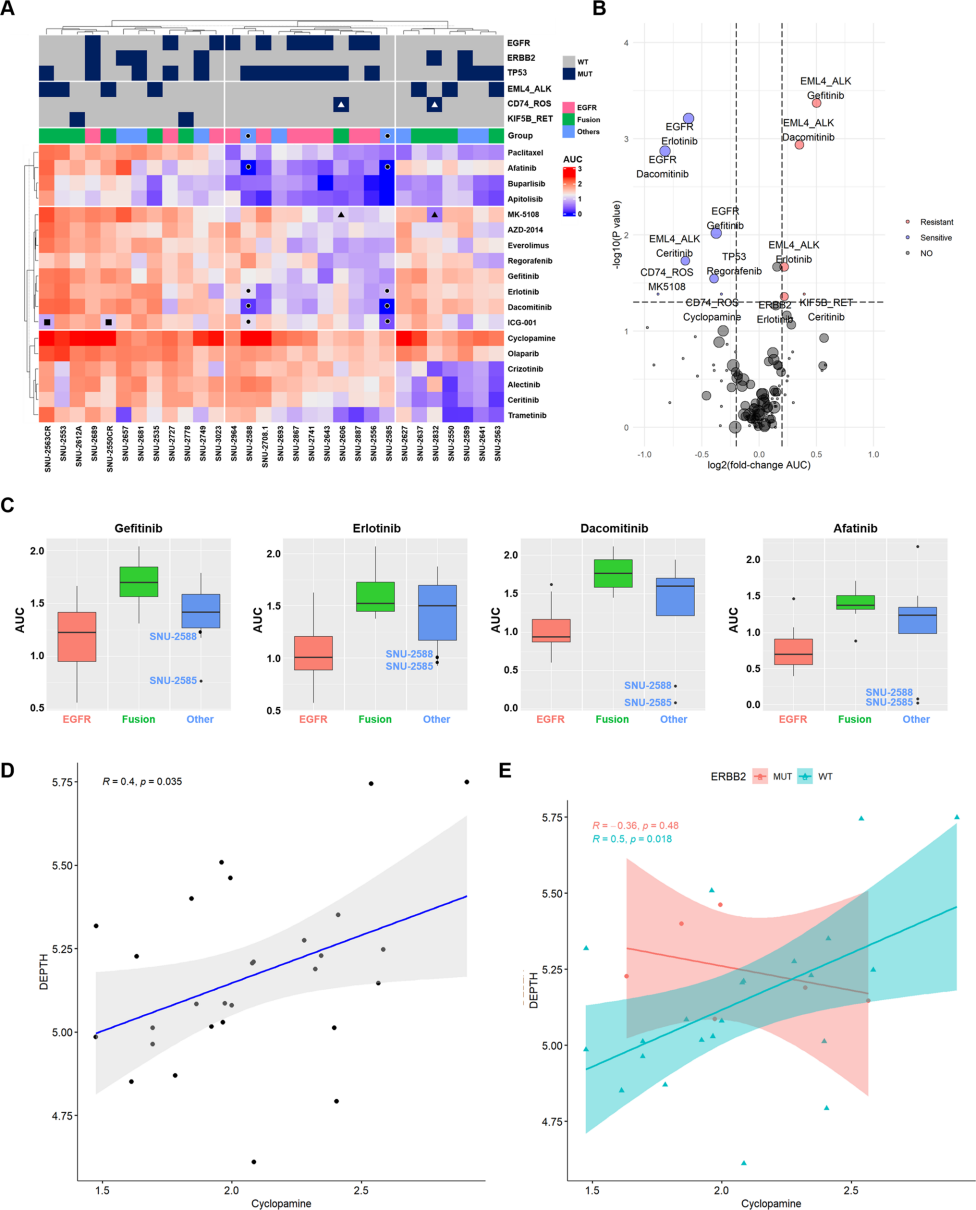
MPE-derived NSCLC Cell Lines Reveal Heterogeneous Drug Responses Caused by Molecular Diversity. (**A**) See also Supplementary Table 5. Heatmap of MPE-derived NSCLC cell lines exhibited heterogeneous distribution of 18 compounds according to their molecular characteristics. The names of compounds are provided on the right. The cell lines and drugs were k-means clustered based on the AUC values across the drug panel. The mutational status of *EGFR*, *ERBB2*, *TP53* and three fusion genes, *EML4-ALK*, *CD74-ROS*, *KIF5B-RET* are specified above the heatmap. (**B)** Volcano plot of gene-drug interaction analysis using Wilcoxon test. Each dot indicated a pair of gene and drug. The size of the circle is proportional to the sample size. When the mutational event was correlated to increased AUC values, the mutation was designated as resistant and colored in red. If the mutation was associated with decreased AUC values, it was assigned as sensitive and colored in blue. The absolute log fold change of AUC value > 0.2 and *p* < 0.05 were considered as significant. (**C)** See also Supplementary Table 6. The box plot of four EGFR-targeting drugs. Inter quartile ranges with the median value are indicated on the box plot. Each group is indicated by representative colors. The names of two outliers (SNU-2585 and SNU-2588) are specified. (**D)** The Pearson correlation coefficient (*R*) with *p* values between the transcriptomic ITH score and AUC of cyclopamine are indicated on the top of the correlation graph. Each dot represents each cell lines. The confidence interval is designated by a shading along with the correlation graph. (**E)** The direct correlation between AUC of cyclopamine and DEPTH is inversed by the mutational status of *ERBB2*. Cell lines with mutant *ERBB2* are separated from the correlation graph and colored in red. The Pearson correlation coefficient (*R*) with *p* values is re-calculated according to the mutational status of *ERBB2*.

As a first validation, k-means clustering of AUC values indicated that both cell lines and drugs were likely grouped with their molecular subtypes. For instance, MPE-derived NSCLC cell lines with *EGFR* mutations such as SNU-2556, SNU-2643, SNU-2708.1, SNU-2741, SNU2867, SNU-2887, SNU-2964 and SNU-3023 as well as EGFR inhibitors including gefitinib, erlotinib and dacomitinib were tightly clustered together. Moreover, MPE-derived NSCLC cell lines with *EML4-ALK* fusion and its targeted drugs including crizotinib, alectinib, and ceritinib were grouped as well (Fig. 5A and Supplementary Table 5).

Among the MPE-derived NSCLC cell lines harboring fusion genes, two cell lines with the *CD74-ROS* fusion gene were selectively sensitive to MK-5108, an Aurora A inhibitor (marked with triangle Fig. 5A). Two crizotinib-resistant sublines displayed insensitivities to other next generation ALK inhibitors including alectinib and ceritinib as well. In accordant with the previous study^21^, ceritinib displayed better responses compared to two other ALK-inhibiting reagents. ICG-001, a β-catenin/CBP interaction inhibitor specifically exhibited good response on both crizotinib-resistant sublines (marked with rectangle Fig.s), which recapitulated the role of the increased nuclear localization of β-catenin caused by the elevated *DKK1* on the crizotinib resistance.

We further integrated the genetic factors of the MPE-derived NSCLC cell lines with 18 drugs using Wilcox ranked sum test (Fig. 5B). Gene-drug interaction analysis not only reconfirmed that EGFR targeted therapy effectively worked on the *EGFR*-mutated cell lines, but also revealed that cell lines harboring the *EML4-ALK* fusion displayed significant insensitivities to EGFR targeted therapy. As indicated earlier, cell lines with *CD74-ROS* fusion were specifically sensitive to MK-5108 as well as cyclopamine.

Although gene-drug interaction analysis demonstrated the heterogeneous drug responses in accordance with the presence of the molecular target, more comprehensive interpretation was achieved by transcriptomic clustering. We performed multivariate analysis of variance (MANOVA) to find which drugs accorded with the three different transcriptional subtypes. MANOVA indicated that EGFR TKIs were significantly associated with the mRNA expression patterns (Supplementary Table 6). For instance, SNU-2585 and SNU-2588 cell lines were free of *EGFR* mutations, yet still positioned adjacent to cell lines with *EGFR* mutations on the transcriptomic analysis. This mRNA expression pattern is reflected by the notably good responses of these cell lines to EGFR TKIs, which suggests that the shape of transcriptomic landscape can be a determinant factor for EGFR-targeting therapy (Fig. 5C). In order to further associate the transcriptomic expression patterns to the drug responses, we then calculated the Pearson correlation coefficient with *p* values between the transcriptional heterogeneity score (DEPTH) and AUCs of each drug. The AUC value of cyclopamine was in direct proportion to the DEPTH score (Fig. 5D). Then, we integrated the genetic factors with the response to cyclopamine in order to further explain outliers. The mutational status of ERBB2 was highly associated with the correlation coefficient of DEPTH score with the AUC of cyclopamine (Fig. 5E). Most of the cell lines with mutated ERBB2 located outside of the confidence interval and had reverse correlation of DEPTH score with the AUC of cyclopamine.

## Discussion

Nearly 20% of lung cancer patients develop malignant pleural effusions (MPEs)^22,23^. MPE is an indicator of the poorer survival rate in lung cancer patients (about 7.5 months) compared to those without effusions (about 12–18 months)^23^. In the tumor-node-metastasis (TNM) staging classification of lung cancer, patients with MPE are categorized as category M1a (stage IV) as it is a sign of metastatic dissemination^24^. Although few studies have focused on malignant tumor cells from pleural fluids, only genomic analysis including mutational aspects and copy number variations was conducted^25–27^. Giuseppe Roscilli et al. integrated the mutational characteristics of human lung adenocarcinoma cells derived from MPEs to the patients chemosensitivity^6^, yet the number of included drugs and patients were not enough to draw solid conclusion.

In this study, we present 28 MPE-derived lung cancer cell lines and two crizotinib resistant sublines that were comprehensively characterized in accordance with multiple drug responses. These in vitro models retained the representative lung cancer mutations in parallel with mutational profiling of other external datasets consisting of lung cancer cell lines (CCLE) as well as tumor samples (TCGA). Our data revealed that MPE-derived lung cancer cell lines involves higher rates of *EGFR* mutations and fusion events compared to the lung adenocarcinoma-originated cell lines. Supporting the notion of distinguished *EGFR* and fusion aberrations, our transcriptomic analysis demonstrated that MPE-derived cell lines were subtyped in parallel with the genetic aberrations. The transcriptomic subtyping of EGFR-mutant NSCLC has been performed in other studies as well^28,29^. Our study has demonstrated that MPE-derived NSCLC cell lines also retain the transcriptional characteristics of EGFR-mutant NSCLC, which were distinctive to NSCLC cell lines with other driver mutations such as *KRAS* or fusion genes.

We suggested that the nuclear localization of β-catenin in MPE-derived NSCLC cell lines through increased DKK1 expression potentially caused crizotinib resistance. The acquire crizotinib resistance has been also associated with both MET amplification and exon 14 skipping mutation in lung cancer^30^. We further inspected if MET aberrations occurred in our two crizotinib sublines as well. We confirmed that MET was clearly amplified in SNU-2550CR cell line compared to its parental SNU-2550 cell line regardless of crizotinib treatment, yet no MET amplification was found in SNU-2563 and SNU-2563CR cell lines. This suggested that MET amplification indeed accounted for acquired crizotinib resistance in SNU-2550 set, and the SNU-2563CR cell lines had different resistance mechanism such as nuclear localization of β-catenin. No MET mutation was found in both resistant sublines.

Our work also enabled the integration of highly prevalent genetic and transcriptional characteristics of MPE-derived lung cancer cell lines to various drug responses. We re-confirmed that targeted therapies, especially EGFR TKIs showed high sensitivities in accordance with the presence of key mutations using Wilcox ranked sum test (Fig. 5B) and MANOVA (Supplementary Table 6) as reported by several previous studies. Remarkably, we identified two EGFR-wild type cell lines (SNU-2585 and SNU-2588) were exceedingly sensitive to EGFR-targeting drugs. Those two cell lines showed even better response to Dacomitinib and Afatinib than EGFR-mutant cell lines (Fig. 5C). Hierarchical clustering analysis using transcriptomic landscape demonstrated that both cell lines were tightly clustered with the EGFR-mutant group (Fig. 3B), which enabled us to conclude that the translational profile can also be a determinant factor for accessing the drug response of targeted therapy. In this perspective, we suggest the downside of using mutation and transcriptomic data separately in clinical diagnosis when predicting MPE-complicated NSCLS patient responses. In analogous with this result, the correlation between the response of MPE-derived NSCLC cell lines to Cyclopamine and transcriptional heterogeneity score (DEPTH) depended on the mutational status of ERBB2 (Fig. 5E).

In conclusion, our work emphasized the significance of integrating the mutational status with transcriptomic subtypes using statistical models such as Pearson correlation coefficient and MANOVA in order for the accurate assessment of drug responses in lung cancer patients with MPE.

## Methods

### Establishment and maintenance of human NSCLC cell lines

The research protocol was reviewed and approved by the institutional review board of the Seoul National University Hospital (IRB No. 1102-098-357). The study was performed in accordance with the Declaration of Helsinki. Written informed consent was obtained from all patients enrolled in this study.

Cell lines were established from malignant pleural effusion (MPE) derived from pathologically proven non-small cell lung cancer (NSCLC). A total of 28 MPE specimens of human NSCLC from 28 different patients who underwent a pleural intervention for the palliation of dyspnea were obtained from Seoul National University Hospital (Seoul, Korea). MPE samples were directly transferred from the operating room to the laboratory for cell culture. Tumor cells were spun down by centrifugation the MPE sample at 300 rpm for 5 min, and re-suspended with Opti-MEM I (GIBCO, CA, USA) supplemented with 5% fetal bovine serum (GE Healthcare Life Sciences, Buckinghamshire, UK) and 1% Penicillin/Streptomycin. Gathered cells were then seeded into T-25cm2 flasks (Corning, NY, USA). Confined-area trypsinization or scraping method was used to attain a pure tumor cell population when stromal cells like mesothelial cells or fibroblasts grew in the initial culture. Established cell lines were sustained in RPMI 1640 medium with 10% fetal bovine serum and 1% (v/v) penicillin and streptomycin (10,000U/ml). Cultures were maintained in humidified incubators at 37 °C in an atmosphere of 5% CO2 and 95% air. The initial passage was assigned when substantial tumor cell growth was detected, and successive passages were given at sub-confluence after trypsinization. When one culture population contains both floating and adherent cells, floating cells were gathered by centrifuging the medium and dispersed by pipetting. All cell lines introduced in this study will be deposited in the Korean Cell Line Bank (http://cellbank.snu.ac.kr) at the initial passage in order to be distributed to researchers worldwide.

### Growth properties and morphology in vitro

To calculate doubling time, the density of 5 × 10^4/ml to 2 × 10^5/ml viable cells were seeded into 96 well cell culture plate with a volume of 200 μl, and cell viability was calculated daily for 7–14 days. Once 10 μl of EZ-Cytox solution (Daeil Lab, Seoul, Korea) was added, the plate was incubated at 37 °C for 2 h, and the optical density of each well was calculated by Multiskan™ GO Microplate Spectrophotometer (Thermo Fisher Scientific, MA, USA) at 450 nm wavelength. Acquired growth rate values were calibrated with GraphPad Prism 5 (GraphPad Software, CA, USA). The morphology of each cell lines was obtained using phase-contrast microscopy. Mycoplasma contamination was checked by the 16S-rRNA-gene-based polymerase chain reaction (PCR) amplification method using e-Myco Mycoplasma PCR Detection Kit (Intron Biotechnology, Gyeonggi, Korea).

### Genomic DNA extraction and DNA fingerprinting analysis

Genomic DNA extraction was performed using QIAamp DNA Mini kit (Qiagen). Genomic DNA extracted from each lung cancer cell line was amplified using an AmpFlSTR identifiler Polymerase Chain Reaction (PCR) Amplification Kit (Applied Biosystems, CA, USA). A single cycle of PCR amplified 15 short tandem repeat markers (CSF1PO, D2S1338, D3S1358, D5S818, D7S820, D8S1179, D13S317, D16S539, D18S51, D19S433, D21S11, FGA, TH01, TPOX and vWA) and an Amelogenin gender-determining marker containing highly polymorphic microsatellite markers. Amplified PCR products were analyzed by an ABI 3500XL Genetic analyzer (Applied Biosystems).

### RNA extraction and reverse transcriptase (RT)-PCR

To obtain RNA, TRIzol (ambion by Invitrogen, CA, USA) was added to cell pellet acquired from cultured lung cancer cell line. After cell lysis was happened, chloroform was added. Then, the sample was vortexed and 4 °C, 12,000 g centrifugation. Each tube containing the sample was divided into aqua phase, interphase, and organic phase. In those aqua phase was transferred into new tube. Isopropanol alcohol was mixed with an equal part of the aqua phase. Mixed sample was centrifuged at 4 °C, 12,000 g after incubated at − 20 °C. RNA was acquired from pellet. The pellet was precipitated by ethanol. RNA was melted in 4th Distilled water containing RNase inhibitor (DEPC). Complementary DNA (cDNA) was synthesized from extracted RNA by reverse transcription kit (Qiagen, MD, USA). Generally measured concentration of RNA was adjusted to 1ug diluted with gDNA wipeout buffer. Extracted RNA concentration was measured nano-spectrometer (Thermo Fisher Scientific, MA, USA). Each 1ug RNA was mixed with RT buffer, RT primer mix, and RTase. Those compounds were heated 42 °C for 30–45 min purposed on annealing, extension. Heated compound was additionally heated 95 °C for 3 min to denature synthetic cDNA. The list of primers we have used are as follows.

**Table:** 

Name	Forward primer sequence	Reverse primer sequence	Annealing temperature (°C)
KIF5B exon 15—RET exon 12 fusion	TAAGGAAATGACCAACCACCAG	GAATTTGGAAAAGTGGTCAAGG	60
KIF5B exon 16—RET exon 12 fusion	GTGAAACG GTGAAACGTTGCAAGCAGTTAG		
EML4 exon 6—ALK exon 20 fusion	GCATAAAGATGTCATCATCAACCAAG	TCTTGCCAGCAAAGCAGTAGTTGGCAGTAGT	60
EML4 exon 13—ALK exon 20 fusion	GTGCAGTGTTTAGCATTCTTGGGG		
EML4 exon 18—ALK exon 20 fusion	GTGGTTTGTTCTGGATGCAGAAACCAGAGATCT		63
EML4 exon 20—ALK exon 20 fusion	GGACATTCCAGCTACATCACACAC		61
CD74 exon 5—ROS1 exon 34 fusion	CCTGAGACACCTTAAGAACACCA	TGAAACTTGTTTCTGGTATCCAA	57
EGFR exon 19 deletion	TTGTGGAGCCTCTTACACCC	AGCAGGTACTGGGAGCCAAT	60
EGFR c.2602 T > G (p.L858R)	ATGTCCGGGAACACAAAGAC	CATCCAGCACTTGACCATGA	60
KRAS exon1 codon12-13	TGACTGAATATAAACTTGTGGTAGTTG	TCGTCCACAAAATGATTCTGAA	59
BRAF c.1799 T > A (p.V600E)	TTGCATCTAAGAGGAAAGAT	GGCCAAAAATTTAATCAGTG	61
ERBB2 c.1963A > G (p.I655V)	GCACCCACTCCTGTGTGGAC	TGCCAAAAGCGCCAGATCCA	62
FGFR4 c.1162G > A (p.G388R) c.991C > T (p.Q331*)	CGAGGCCAGGTATACGGACA	CAAAGGCCTCTGCACGTACT	63
TP53 exon4	TCCCCTGCCCTCAACAAGAT	AACCTCCGTCATGTGCTGTG	60
TP53 exon7	CTGGTGGTGCCCTATGAGCC	AGGAGCTGGTGTTGTTGGGC	61
TP53 exon8	CCTCTTTCCTAGCACTGCCC	CAAATGCCCCAATTGCAGGT	60
ALK kinase domain	GGAGGTGTATGAAGGCCAGG	TCGGAGGAAGGACTTGAGGT	61
ALK kinase domain (S1206Y, G1269A mutation target)	ACCTCAAGTCCTTCCTCCGA	CACTTAACTGGCAGCATGGC	61
ROS1 kinase domain	CTGCCTTCCCTCGGGAAAAA	GCTGCCAGATCCCTGTGAAT	63
β-actin	GACCACACCTTCTACAATGA	GCATACCCCTCGTAGATGGG	60

### Complementary DNA synthesis

For cDNA synthesis, QuantiTect Reverse Transcription Kit (Qiagen) was used. One microgram of total RNA, 2 μL of gDNA Wipeout Buffer, and RNase free water up to 14 μL were mixed together and incubated at 42 °C for 2 min. The mixture was mixed with Quantiscript RT Buffer, RT Primer Mix, and Quantiscript Reverse Transcriptase and incubated at 42 °C for 45 min. Then, the mixture was incubated at 95 °C for 2 min and cooled down to room temperature.

### Sanger sequencing

PCR product was precipitated by 5% sodium acetate buffer (Sigma-Aldrich) and 95% ethanol mixed solution. Then washed product was set on ice for 10 min and centrifuged at 4 °C, 14,000 rpm. Supernatant was discarded and the product was rinsed this time by 70% ethanol and centrifuged 14,000 rpm. Supernatant was discarded then the products were dried using vacuum concentrator (Eppendorf). 10 μL of distilled water was added to dilute precipitated sample. When the product is all diluted in distilled water, cyclic PCR was carried out. Two separate mixtures for forward and reverse sequences were made where they each include 5X sequencing buffer (Applied Biosystems), Big Dye (Applied Biosystems), forward or reverse primer, distilled water, and product from the previous step. Cyclic PCR was carried out with denaturation step at 96 °C, annealing temperature at 55 °C, and elongation at 60 °C for 25 cycles. The cyclic PCR product was then precipitated with 5% sodium acetate buffer and 95% ethanol mixed solution and set on ice for 10 min then it was centrifuged at 4 °C and supernatants were carefully discarded and the final product was dried using the vacuum concentrator. 10 μL Hi-Di formamide (Applied Biosystems) was added to dilute the dried product. This final product was transferred to 96 well PCR plate and denatured at 95 °C for 2 min before taken to 3500xL Genetic Analyzer (Applied Biosystems) for sequencing.

### CCP analysis, targeted gene sequencing

CCP (Comprehensive Cancer Panel) is technology using Ion Ampliseq (Thermofisher Scientific). CCP panel could detect 409 genes mutations on exomes. There are various mutation types about driver-genes. According to the manufacturer’s instructions as described, gDNA was extracted from cell pellet specimen using QIAamp DNA Mini kit. Quantity and quality of obtained DNA samples was evaluated using the Qubit fluorometer and the Qubit dsDNA HS assay kit (Life Technologies, Gent, Belgium). AmpliSeq library was assembled with NEBNext® Ultra DNA Library Prep Kit for Illumina (NEB #E7370S/L) and the Ion AmpliSeqTM Library Kit 2.0 (Life Technologies, Part #4480441 Rev. 5.0). And then, the amplicons were purified with AMPure XP reagent (Beckman Coulter, Brea, CA, USA). The products were processed in end repair by NEBNext® End Prep kit (NEB #E7442). NEBNext® Multiplex Oligos for illumina (NEB #E7335 or NEB #E7500) were ligated with DNA ligase. Adapter-ligated products were then purified by AMPure XP reagent (Beckman Coulter, Brea, CA, USA), and PCR-amplified for a total of 7 cycles. The complete library was purified by AMPure XP reagent (Beckman Coulter) and GeneRead size selection kit(Qiagen). Size and concentration of the library were analyzed by Agilent 2100 BioAnalyzer and Agilent BioAnalyzer DNA High-Sensitivity LabChip (Agilent Technologies). The amplified libraries’s quality was proved by capillary electrophoresis (Bioanalyzer, Agilent). Qualified libraries were mixed in index tagged in equimolar amounts of the pool, after QPCR that was taken by using SYBR Green PCR Master Mix (Applied Biosystems). Sequencing was completed using an Illumina NextSeq 500 system following referred protocols for 2 × 150 bp sequencing.

### Drug sensitivity test

2 × 10^5 to 4 × 10^5viable cells from each cell line were seeded into well of 96 well plate in triplicate to measure drug sensitivity of 18 compounds. After 72 h-incubation at 37 °C, 10ul EZ-Cytox solution was added to well of each seeded lung cancer cells. After 2 h-incubation at 37 °C, optical density of EZ-Cytox-treated cells was calculated by Multiskan™ GO Microplate Spectrophotometer (Thermo Fisher Scientific). These steps were repeated in triplicate. The half maximal effective concentration (EC50) were measured by GraphPad Prism 5 (GraphPad Software). The maximum concentration and solvent are as follows.

**Table:** 

Drugs	Company	Cat no.	Solvent	Max conc. (uM)
Afatinib	Selleckchem	Cat# S1011	DMSO	50
Alectinib	Selleckchem	Cat# S2762	DMSO	10
Apitolisib	Selleckchem	Cat# S2696	DMSO	50
Buparlisib (BKM120)	Selleckchem	Cat# S2247	DMSO	100
Ceritinib	Selleckchem	Cat# S7083	DMSO	10
Crizotinib	Selleckchem	Cat# S5190	DMSO	10
Cyclopamine	Selleckchem	Cat# S1146	DMSO	50
Dacomitinib	Selleckchem	Cat# S2727	DMSO	10
Erlotinib Hydrochloride	Selleckchem	Cat# S1023	DMSO	100
Everolimus	Selleckchem	Cat# S1120	DMSO	100
Gefitinib	Selleckchem	Cat# S1025	DMSO	100
ICG-001	Selleckchem	Cat# S2662	DMSO	100
MK-5108	Selleckchem	Cat# S2770	DMSO	100
Olaparib	Selleckchem	Cat# S1060	DMSO	50
Paclitaxel	Selleckchem	Cat# S1150	DMSO	50
Regorafenib	Selleckchem	Cat# S1178	DMSO	100
Trametinib	Selleckchem	Cat# S2673	DMSO	50
Vistusertib (AZD2014)	Selleckchem	Cat# S2783	DMSO	5

### Protein extraction and Western blotting

Cultivated cells that had full confluency were harvested with cell scrapper. Cell pellet was treated by EzRIPA buffer (ATTO Co., Tokyo, Japan) after washed by cool PBS. Whole protein was extracted by this step. Protein concentration of each cell line was determined by SMART micro BCA protein assay kit (Intron biotechnology). Proteins that fixed into equal concentration were loaded on a 4–12% Bis–Tris gel (Invitrogen) at 70 V for 3 h and then proteins on loaded gel were transferred to a PVDF membrane (Invitrogen) by electro-blotting in condition under a constant current of 80 mA at 4 °C overnight. Proteins of transferred membrane was blocked by incubating in 1.5% to 2.0% skim milk and 0.05% Tween 20-TBS buffer including 1 mM MgCl2 for an hour at room temperature. Primary antibodies were used against DKK1, β-catenin, E-cadherin, and Vimentin and β-actin. Mouse or rabbit IgG 2nd antibody (Jackson Immunoresearch, PA, USA) (1:5000) conjugated with peroxidase that matched with used 1st antibody was added to membrane. After chemiluminescent working solution, WESTZOLTM (Intron biotechnology) was treated to the membrane, the membrane was exposed to Fuji RX film (Fujifilm, Tokyo, Japan) for 1–5 min. The raw and unprocessed blotting images are provided with supplementary data file. The blotting image for DKK1 was closely cropped near the membrane edges, and we provide replicates performed as well. The MET protein level was confirmed with ChemiDoc Touch Imaging System (Bio-Rad Laboratiores, CA, USA) using consecutive exposing mode with 1-min interval for 5 min.

**Table:** 

Target	2nd species	Company	Cat no.	Dilution
E-Cadherin	Rabbit	Abcam	Cat# 1702-1, RRID:AB_562059	1:1000
Vimentin	Rabbit	Abcam	Cat# ab63379, RRID:AB_1524552	1:1000
DKK1	Mouse	Abcam	Cat# ab56905, RRID:AB_941302	1:1000
MET	Mouse	Abcam	Cat# ab51067, RRID:AB_880695	1:1000
β-catenin	Rabbit	Abcam	Cat# ab53089, RRID:AB_868701	1:1000
β-actin	Mouse	Santa Cruz Biotechnology	Cat# sc-130301, RID:AB_2223360	1:2000

### Immunocytochemistry

Cells were seeded on chambered coverglass (Thermo Fisher Scientific, MA, USA) with a desirable cell confluency. The chambered coverglass was designed to be hydrophilic and no ECM component was treated before seeding. 72 h after cell seeding, cells were washed with cold DPBS for 15 min three times. Then, cells were fixed and permeabilized with BD Cytofix/Cytoperm™ (BD science, CA, USA). After cells were washed with washing solution (BD science), DPBS containing 2% FBS (GE Healthcare Life Sciences, Buckinghamshire, UK) was applied for an hour for blocking. After cells were washed with cold DPBS β-catenin antibody (1:500, Abcam, Cambridge, United Kingdom) diluted in 0.05% of PBS.T was applied for a 1.5 h in room temperature. Thereafter, cells were washed with 0.05% of PBS.T, and Alexa 488 and Alexa 568 secondary antibodies (Thermo Fisher Scientific, MA, USA) diluted in 0.05% of PBS.T were applied for an hour in room temperature. 1 × DAPI and Rhodamine-conjugated Phalloidin (Sigma-Aldrich, MO, USA) were diluted in distilled water and applied for 30 min in room temperature. The cells were washed with DPBS three times, and pictured under confocal microscope. LSM800 Confocal Laser Scanning Microscope and ZEN software (Carl Zeiss, Oberkochen, Germany) was used to examine cells. Diverse magnifications were used for different growth patterns and sizes of cells. The intensity of each channel was fixed for the comparison of target protein expression between samples. Digital resolution, scan speed and the number of pictures averaged were set to 1024 × 1024, 40 s per one channel, and 8 pictures respectively. The pictures were focused on the very bottom of the fixed cells for investigating protruding region of cell colonies and the location of β-catenin.

### RNA sequencing

Total RNA was isolated from cell lysate using Trizol (Qiagen) and Qiagen RNeasy Kit (Qiagen). Sequencing libraries were prepared using the Illumina TruSeq Stranded Total RNA Library Prep Kit. Fifty-one million reads were obtained from the cell lysates. Following base-calling and alignment with the Tuxedo Suite, rejected reads were analyzed using FusionMap, ChimeraScan, and Defuse with default parameters for RNA and alignment to GRCh37.72. The output was filtered to include in-frame fusions, with at least one rescued read and two unique seed reads, and exclude known, recurrent artifacts.

### Whole exome sequencing

SureSelect sequencing libraries were prepared according to manufacturer’s instructions (Agilent sureselect all Exon kit 50 Mb) using the Bravo automated liquid handler. Three micrograms of genomic DNA were fragmented to a median size of 150 bp using the Covaris-S2 instrument (Covaris, Woburn, MA). The adapter ligated DNA was amplified by PCR, and the PCR product quality was assessed by capillary electrophoresis (Bioanalyzer, Agilent). The hybridization buffer and DNA blocker mix were incubated for 5 min at 95 °C and then for10 minutes at 65 °C in a thermal cycler. The hybridization mixture was added to the bead suspension and incubated for 30 min at RT while mixing. The beads were washed, and DNA was eluted with 50 ml SureSelect elution buffer (Agilent). The flow cell loaded on HISEQ 2500 sequencing system (Illumina).

### Statistical analysis

Statistical analysis was performed using R program version 3.3.1 (R Foundation for Statistical Computing, Vienna, Austria) with various packages including maftools, PerformanceAnalytics, survminer, survival, iplot, gplot, and lattice. Fisher’s exact test was used to analyze GO analysis of various genes. A multivariate analysis of variance (MANOVA) model was applied to the drug response data matrix with various factors such as the mutational status and the three different transcriptional subtypes. Approximate F value, p-value and Pillai’s trace score were obtained for each of the factors/drug pairs. A value of *p* < 0.05 was considered statistically significant. For hierarchical cluster analysis on a set of dissimilarities, each object was assigned to its own cluster, which an algorithm proceeds through iteratively. Two of the most similar clusters are joined at each stage until there is a single cluster. Distances between clusters are recomputed at each stage by the Lance–Williams dissimilarity update formula according to the particular clustering method being used. Clustering methods include: Ward's minimum variance method, complete linkage method, k-means method, and single linkage method.

## Supplementary Information

Supplementary Information 1.

Supplementary Table S3.

Supplementary Table S5.
