# Supplementary material for: Culture and multiomic analysis of lung cancer patient-derived pleural effusions revealed distinct druggable molecular types
Source: Sci Rep. 2022 Apr 15;12:6345. doi: 10.1038/s41598-022-10318-5 (PMC9012760; doi:10.1038/s41598-022-10318-5)
Supplement: Supplementary file 1 — Supplementary Information 1. [file 41598_2022_10318_MOESM1_ESM.docx]

**Supplementary Information**

**Culture and Multiomic Analysis of Lung Cancer Patient-Derived Pleural Effusions Revealed Distinct Druggable Molecular Types**

Ha-Young Seo^1,3,†^, Soon-Chan Kim^1,2,3,4,†^, Woo-lee Roh^1^, Young-Kyoung Shin^1^, Soyeon Kim^3^, Dong-Wan Kim^3,5^, Tae Min Kim^3,5^, Ja-Lok Ku^1,2,3,4,6,*^

^1^Korean Cell Line Bank, Laboratory of Cell Biology, Cancer Research Institute, Seoul National University College of Medicine, Seoul 03080, Korea

^2^Department of Biomedical Sciences, Seoul National University College of Medicine, Seoul 03080, Korea

^3^Cancer Research Institute, Seoul National University, Seoul 03080, Korea

^4^Ischemic/Hypoxic Disease Institute, Seoul National University College of Medicine, Seoul, 03080, South Korea

5Department of Internal Medicine, Seoul National University Hospital, Seoul 03080, Korea

^6^Lead Contact


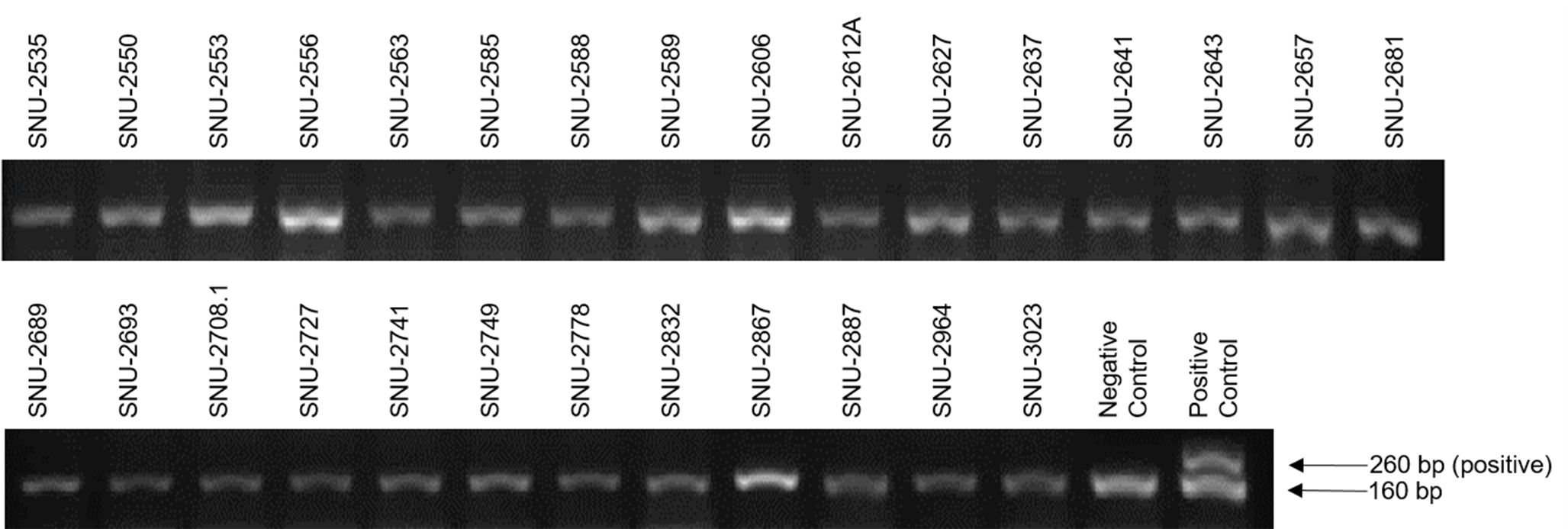


**Supplementary Figure 1.** **Related to Figure 1.** Mycoplasma test of 28 MPE-derived cell lines. All cell lines were free from mycoplasma contamination.


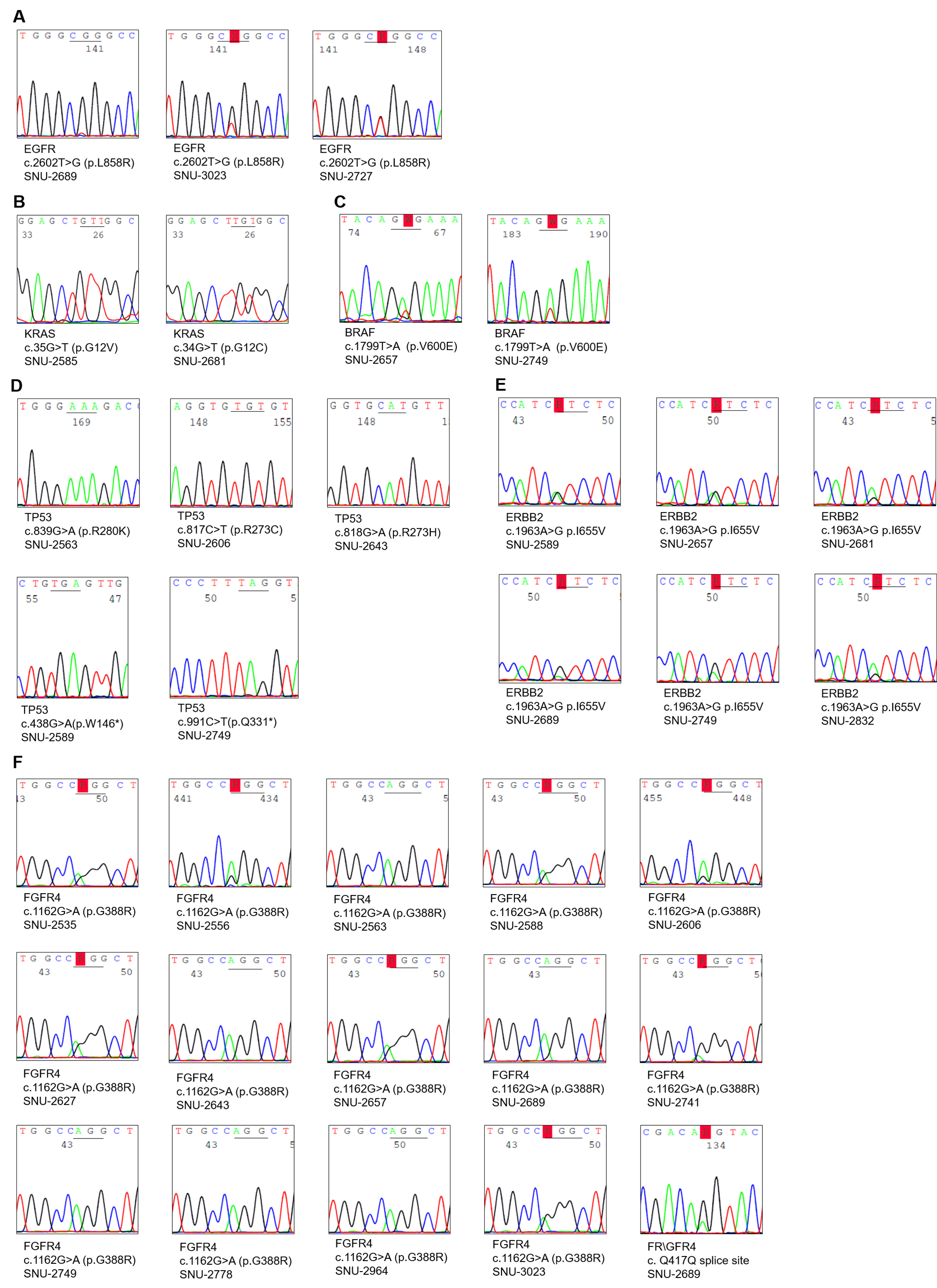


**Supplementary Figure 2A-F.** **Related to Table S2.** Sanger sequencing confirmed the presence of mutations that were detected from the targeted sequencing to minimize false positive sequencing errors.


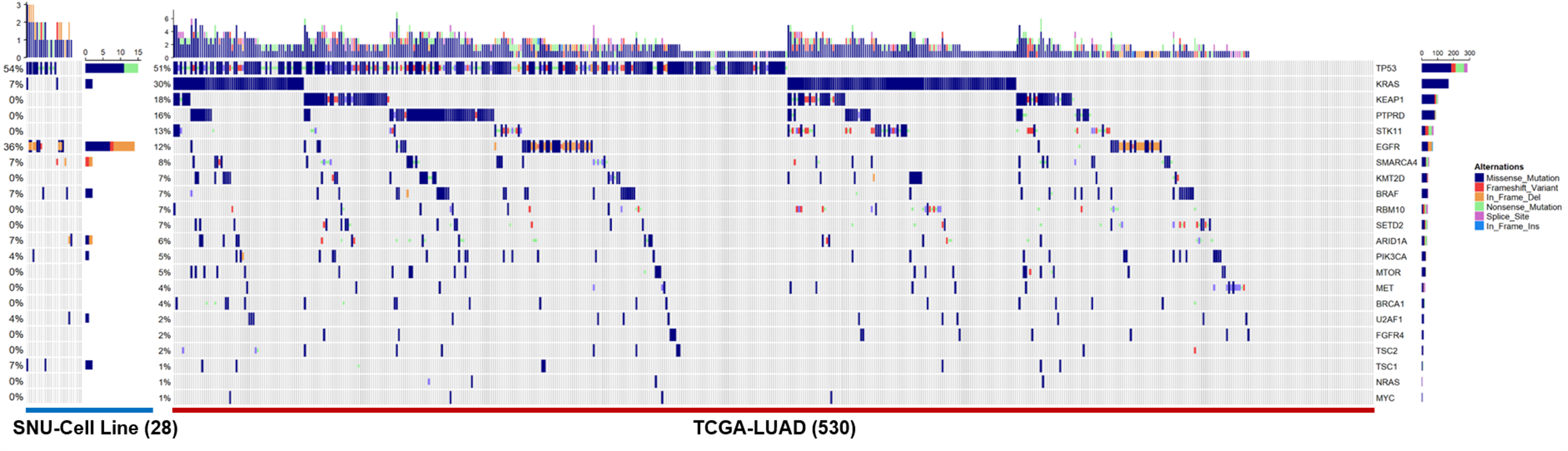


**Supplementary Figure 3.** **Related to Figure 2A.** The mutational profiles of MPE-derived lung cancer cell lines (*n* = 28) were compared to TCGA cohort (*n* = 530). The mutational proportions are calculated within each cohort and indicated on the left side of the plot. The total number of mutations within each cohort is specified on the right side of the plot. The total number of mutations within each cell lines or tissue samples is listed on the top of the plot. The different types of mutations are marked with representative colors.


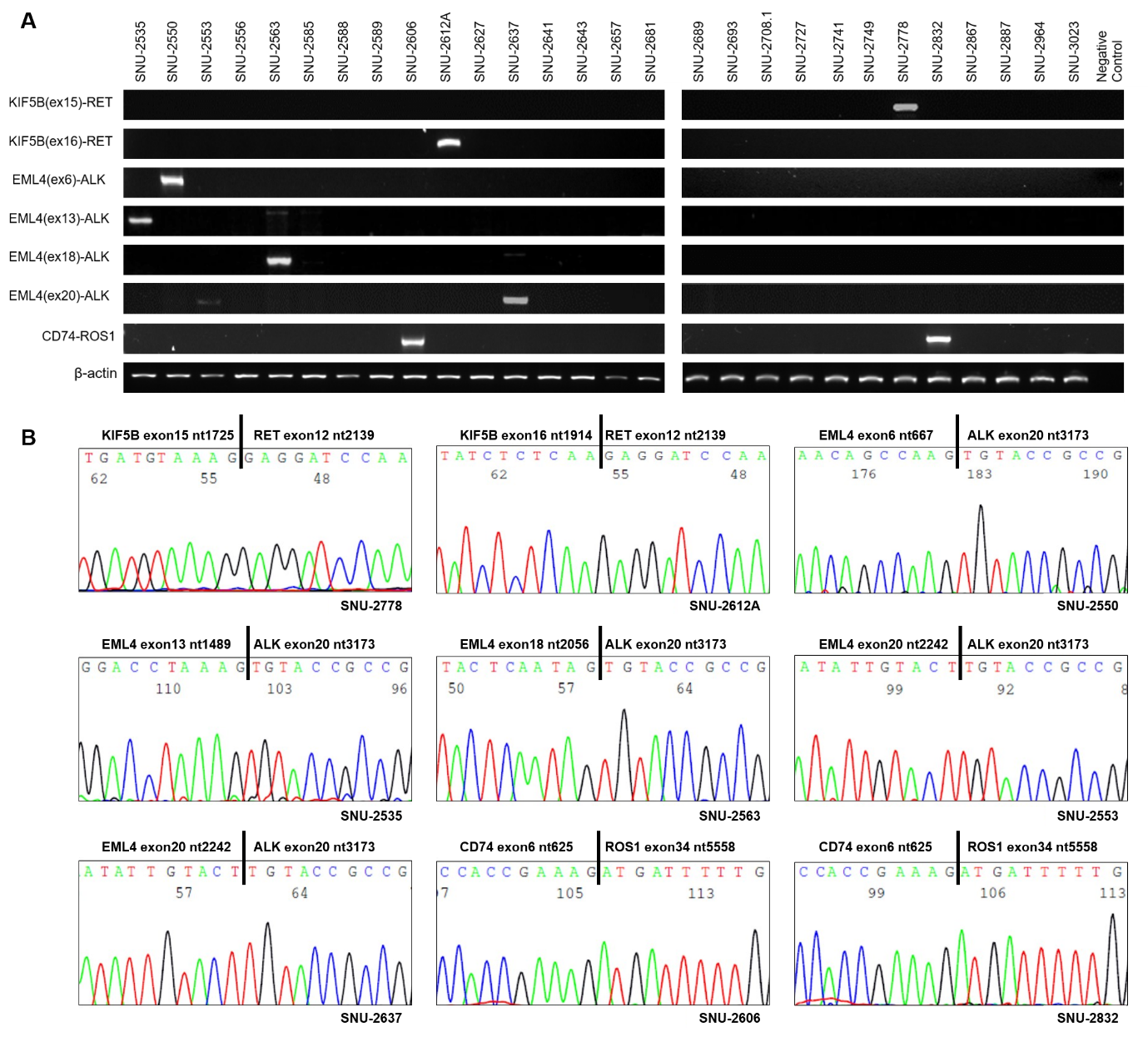


**Supplementary Figure 4. Related to Figure 4.** Detection of fusion genes by RT-PCR and sanger sequencing. **A.** Specific primers targeting break junctions of each fusion gene is used to detect various fusion genes. **B.** Detected fusion genes were validated with sanger sequencing.


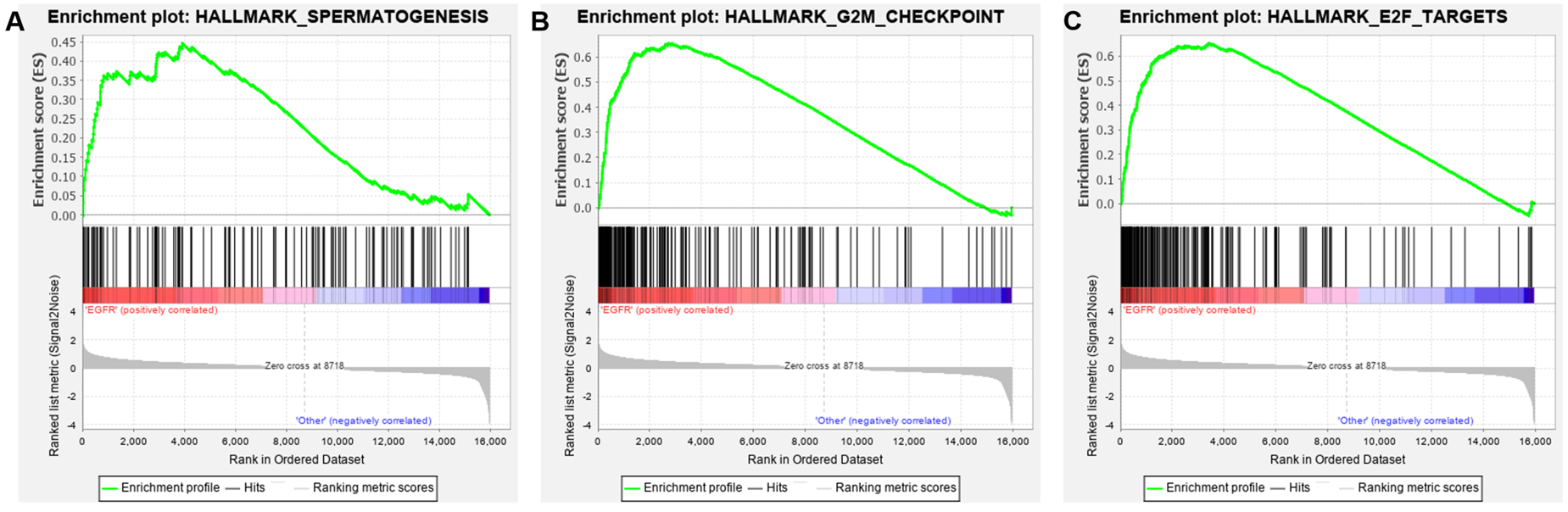


**Supplementary Figure 5. Related to Figure 3.** GSEA analysis using EGFR-mutant CCLE database revealed that spermatogenesis pathway is also upregulated in EGFR-mutant groups in larger dataset.


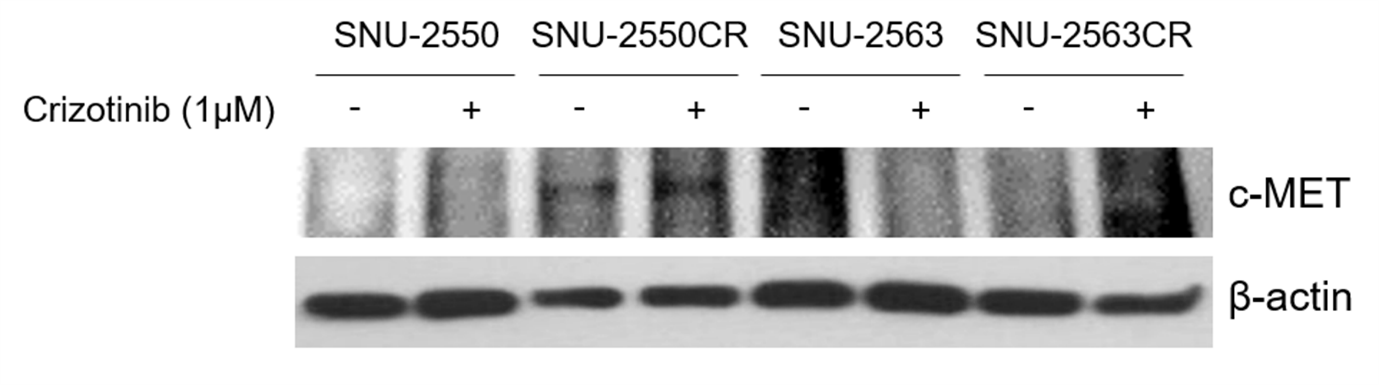


**Supplementary Figure 6. Related to Figure 4.** Confirmation of MET amplification in crizotinib resistant sublines. The basal level of MET was amplified in SNU-2550CR cell line regardless of crizotinib treatment, and no MET amplification was found in SNU-2563 set.

| **Cell Line** | **D8S1179** | **D21S11** | **D7S820** | **CSF1PO** | **D3S1358** | **TH01** | **D13S317** | **D16S539** | **D2S1338** | **D19S433** | **Vwa** | **TPOX** | **D18S51** | **Amelogenin** | **D5S818** | **FGA** |
| --- | --- | --- | --- | --- | --- | --- | --- | --- | --- | --- | --- | --- | --- | --- | --- | --- |
| SNU-2535 | 13,15 | 28,29 | 11 | 11 | 14,15 | 7,9.3 | 9,11 | 9,11 | 20,23 | 10.2,14 | 16,17 | 11 | 15 | X | 10,12 | 24,27 |
| SNU-2550 | 14,15 | 29,30 | 8,12 | 10 | 15 | 9 | 8,11 | 9 | 23,24 | 12,15,15.2 | 14,18 | 11 | 8,17 | X | 11,12 | 19 |
| SNU-2553 | 12,13 | 30.2,31 | 9,11 | 11,12 | 16 | 7 | 8 | 11 | OL,23 | 13,14 | 14,15 | 8,9 | 16 | X,Y | 9,11 | 21,24 |
| SNU-2563 | 11,15 | 28,29 | 10,11 | 10 | 15 | 7,9.3 | 11,13 | 12,13 | 18,23 | 13.2,15 | 17,18 | 8,11 | 15 | X,Y | 10,11 | 22,23 |
| SNU-2556 | 11,14 | 29,32.2 | 9,10 | 10,11 | 16 | 9 | 12,13 | 9 | 20,26 | 14,14.2 | 14 | 8,11 | 15 | X,Y | 11 | 23,24 |
| SNU-2585 | 12,16 | 31,31.2 | 10,12 | 12 | 16 | 7 | 9,10 | 9,12 | 19,20 | 13 | 18 | 8,9 | 13,18 | X,Y | 10,11 | 22 |
| SNU-2588 | 10,13 | 32.2 | 10,12 | 10,11 | 17 | 7,9 | 8,12 | 12 | 24,25 | 13.2,15 | 18 | 8 | 13,17 | X,Y | 11,13 | 24 |
| SNU-2589 | 14,16 | 30 | 8,12 | 12,13 | 15 | 6,8 | 11,12 | 9 | 17,20 | 13,17.2 | 17 | 8,11 | 13,14 | X | 11,12 | 21,22 |
| SNU-2606 | 13 | 29 | 9,12 | 12,13 | 15 | 7,9 | 9 | 8,9 | 24,26 | 13,15.2 | 17,19 | 8,11 | 16 | X | 9,13 | 21,26 |
| SNU-2612A | 13,14 | 30 | 8,10 | 10 | 15,16 | 9 | 8,12 | 9,13 | 19,21 | 13,14 | 18 | 8,10 | 13,16 | X,Y | 9 | 18,21 |
| SNU-2627 | 10,11 | 30,32.2 | 10,11 | 12,13 | 17 | 7,9 | 12 | 12 | 25 | 13,14.2 | 17,19 | 8,11 | 21 | X | 11,12 | OL |
| SNU-2637 | 12,13 | 30,31.2 | 8,9 | 9,13 | 16,17 | 9 | 9,11 | 9 | 18,23 | 14.2,15.2 | 18,19,20 | 8 | 13,21 | X,Y | 11 | 22,25 |
| SNU-2641 | 13 | 30,31.2 | 11,13 | 12,13 | 15 | 9 | 8,10 | 10,13 | 17,23 | 13 | 16,17 | 8,11 | 14,15 | X,Y | 11 | 17,21 |
| SNU-2643 | 13 | 29,31 | 8,11 | 10,12 | 15,17 | 6,7 | 8 | 12 | 19,24 | 13.2,15 | 14,17 | 8,11 | 20 | X | 11 | 22,24 |
| SNU-2657 | 10,12 | 29 | 9,10 | 11,12 | 14 | 6,7 | 8 | 9,12 | 24 | 13,15 | 16 | 11,12 | 13,22 | X | 11,12 | 22,23 |
| SNU-2681 | 11,12 | 29.2,30 | 11 | 10,12 | 15 | 9 | 10 | 12 | 17,23 | 14,15.2 | 17 | 8 | 13 | X,Y | 9,10 | 21 |
| SNU-2689 | 11,14 | 30 | 11 | 12,14 | 15,17 | 6 | 11 | 9 | 22 | 14.2,15.2 | 17 | 11 | 14,17 | X | 10,11 | 20 |
| SNU-2693 | 14,15 | 29,30 | 8,10 | 10 | 15,18 | 9 | 13 | 11,12 | 18,24 | 13 | 16 | 10,11 | 13 | X | 10 | 27 |
| SNU-2708.1 | 14 | 29,30 | 8,10 | 10,11 | 18 | 9 | 11 | 9,12 | 24 | 14 | 17 | 9 | 17 | X,Y | 11 | 20,23 |
| SNU-2727 | 11 | 29,31.2 | 11,14 | 10 | 16 | 6,8 | 9 | 9 | 19 | 13,14 | 14 | 8 | 17 | X | 11 | 23 |
| SNU-2741 | 14 | 30,32.2 | 8,12 | 9,12 | 15 | 7,9 | 8 | 9,11 | 23 | 10.2,14 | 17 | 8 | 14 | X | 10,11 | 22 |
| SNU-2749 | 14 | 30 | 9,12 | 12 | 16,17 | 6,7 | 10,12 | 10,12 | 18 | 13,14 | 14,18 | 8,11 | 12 | X,Y | 11 | 22,23 |
| SNU-2778 | 13,16 | 32.2 | 12 | 10,11 | 15,17 | 8 | 8 | 9,10 | 23,26 | 14,15.2 | 14 | 8 | 20 | X | 10,12 | 22,23 |
| SNU-2832 | 15,16 | 30,31 | 8,11 | 7,11 | 15,19 | 6 | 10,11 | 9,13 | 18,24 | 14 | 14 | 8 | 14,15 | X | 11,12 | 21,22 |
| SNU-2867 | 15 | 29,30 | 9,10 | 12 | 15,16 | 6,9 | 8,11 | 9 | 23 | 13,14 | 20 | 11 | 16 | X,Y | 11 | 24 |
| SNU-2887 | 13 | 29,31 | 10,11 | 12 | 17 | 7 | 12 | 10 | 23 | 16.2 | 18 | 8 | 14 | X | 11 | 24,25 |
| SNU-2964 | 13,15 | 29.2,30 | 10,11 | 10,12 | 15,16 | 7,9 | 12 | 9,11 | 17 | 14 | 17 | 8,9 | 16 | X | 9,12 | 21,22.2,23.2 |
| SNU-3023 | 15,16 | 31 | 8,11 | 12 | 14 | 6,9.3 | 12 | 11 | 19,25 | 14,15 | 14,16 | 8 | 15 | X | 12 | 23,24 |

**Supplementary Table 1.** STR profiles of 28 human lung cancer cell lines.

| **Name** | **BRAF** | **EGFR** | **ERBB2** | **FGFR4** | **KRAS** | **TP53** |
| --- | --- | --- | --- | --- | --- | --- |
| SNU-2535 |  |  |  | Ggg>Agg(G388R) |  |  |
| SNU-2550 |  |  |  |  |  |  |
| SNU-2553 |  |  |  |  |  |  |
| SNU-2563 |  |  |  |  |  | aGa>aAa(R280K) |
| SNU-2556 |  |  |  | Ggg>Agg(G388R) |  |  |
| SNU-2585 |  | Aat>Gat(N528D) |  |  | gGt>gTt(G12V) |  |
| SNU-2588 |  |  |  | Ggg>Agg(G388R) |  |  |
| SNU-2589 |  | cGg>cAg(R675Q) | Atc>Gtc(I655V) |  |  | tgG>tgA(W146*) |
| SNU-2606 |  |  |  | Ggg>Agg(G388R) |  | Cgt>Tgt(R273C) |
| SNU-2612A |  |  |  |  |  |  |
| SNU-2627 |  |  |  | Ggg>Agg(G388R) |  |  |
| SNU-2637 |  |  |  |  |  |  |
| SNU-2641 |  |  |  |  |  |  |
| SNU-2643 |  |  |  | Ggg>Agg(G388R) |  | cGt>cAt(R273H) |
| SNU-2657 | gTg>gAg(V600E) |  | Atc>Gtc(I655V) | Ggg>Agg(G388R) |  |  |
| SNU-2681 |  |  | Atc>Gtc(I655V) |  | Ggt>Tgt(G12C) | cGt>cTt(R110L) |
| SNU-2689 |  | cTg>cGg(L858R) | Atc>Gtc(I655V) | Ggg>Agg(G388R), caG>caA(Q417Q) |  |  |
| SNU-2693 |  |  |  |  |  |  |
| SNU-2708.1 |  | cTc>cAc(L1017H), aaggaattaagagaagca>aaa (ELREA701del) |  |  |  |  |
| SNU-2727 |  |  |  |  |  |  |
| SNU-2741 |  |  |  | Ggg>Agg(G388R) |  |  |
| SNU-2749 | gTg>gAg(V600E) |  | Atc>Gtc(I655V) | Ggg>Agg(G388R) |  | Cag>Tag(Q331*) |
| SNU-2778 |  |  |  | Ggg>Agg(G388R) |  |  |
| SNU-2832 |  |  | Atc>Gtc(I655V) |  |  |  |
| SNU-2867 |  |  |  |  |  |  |
| SNU-2887 |  |  |  |  |  |  |
| SNU-2964 |  |  |  |  |  |  |
| SNU-3023 |  | cTg>cGg(L858R) |  | Ggg>Agg(G388R) |  |  |

**Supplementary Table 2.** **See also Figure S2A-F.** Driver Mutation Profiles Detected by Targeted Gene Sequencing.

| **Upregulated in class** | **GeneSet** | **Enrichment Score (ES)** | **Normalized Enrichment Score (NES)** | **Nominal p-value** | **FDR q-value** | **FWER p-Value** |
| --- | --- | --- | --- | --- | --- | --- |
| Fusion | HALLMARK_INFLAMMATORY_RESPONSE | 0.404 | 1.579 | 0.005 | 0.126 | 0.272 |
| Fusion | HALLMARK_HYPOXIA | 0.465 | 1.740 | 0.007 | 0.128 | 0.099 |
| Fusion | HALLMARK_TNFA_SIGNALING_VIA_NFKB | 0.497 | 1.683 | 0.020 | 0.106 | 0.147 |
| Fusion | HALLMARK_ANGIOGENESIS | 0.550 | 1.607 | 0.038 | 0.130 | 0.228 |
| EGFR | HALLMARK_SPERMATOGENESIS | 0.402 | 1.494 | 0.021 | 0.909 | 0.419 |
| EGFR | HALLMARK_E2F_TARGETS | 0.537 | 1.427 | 0.181 | 0.710 | 0.517 |
| EGFR | HALLMARK_G2M_CHECKPOINT | 0.483 | 1.338 | 0.211 | 0.772 | 0.636 |
| EGFR | HALLMARK_APICAL_SURFACE | 0.371 | 1.245 | 0.149 | 0.913 | 0.748 |

**Supplementary Table 4.** **See also Figure 3C,D.** Genes with high enrichment scores for each signaling pathway

**Supplementary Table 6. See also Figure 6C.** Genes with high enrichment scores for each signaling pathway

| Drug | Sum Sq | Mean Sq | F value | Pr(>F) | Signif.code |
| --- | --- | --- | --- | --- | --- |
| Erlotinib | 1.452 | 0.72597 | 8.2509 | 0.001874 | ** |
| Crizotinib | 0.5217 | 0.26083 | 1.6092 | 0.2209 |  |
| Everolimus | 0.26093 | 0.13046 | 1.1003 | 0.349 |  |
| Paclitaxel | 0.397 | 0.19848 | 0.9423 | 0.4037 |  |
| Trametinib | 0.1647 | 0.082355 | 0.3697 | 0.6948 |  |
| ICG001 | 0.53284 | 0.266418 | 2.8899 | 0.07507 | . |
| Alectinib | 0.6886 | 0.34429 | 1.909 | 0.1701 |  |
| Ceritinib | 0.8733 | 0.43666 | 2.6349 | 0.09237 | . |
| Dacomitinib | 2.275 | 1.13752 | 5.6931 | 0.009474 | ** |
| Gefitinib | 1.1183 | 0.55915 | 5.941 | 0.008017 | ** |
| Afatinib | 1.6447 | 0.82233 | 3.8542 | 0.03535 | * |
| Regorafenib | 0.16119 | 0.080595 | 0.9716 | 0.3929 |  |
| Buparlisib | 0.1949 | 0.097467 | 0.7224 | 0.4958 |  |
| Apitolisib | 0.0736 | 0.036821 | 0.2485 | 0.782 |  |
| Cyclopamine | 0.0603 | 0.030135 | 0.2231 | 0.8017 |  |
| AZD2014 | 0.01455 | 0.007275 | 0.097 | 0.9079 |  |
| MK5108 | 0.1649 | 0.082474 | 0.6171 | 0.5479 |  |
| Olaparib | 0.05902 | 0.029509 | 0.5503 | 0.5839 |  |

Signif. codes:  ‘***’ < 0.001, ‘**’ < 0.01, ‘*’ < 0.05, ‘.’ < 0.1

**The original, unprocessed versions of gels and blots.**

**
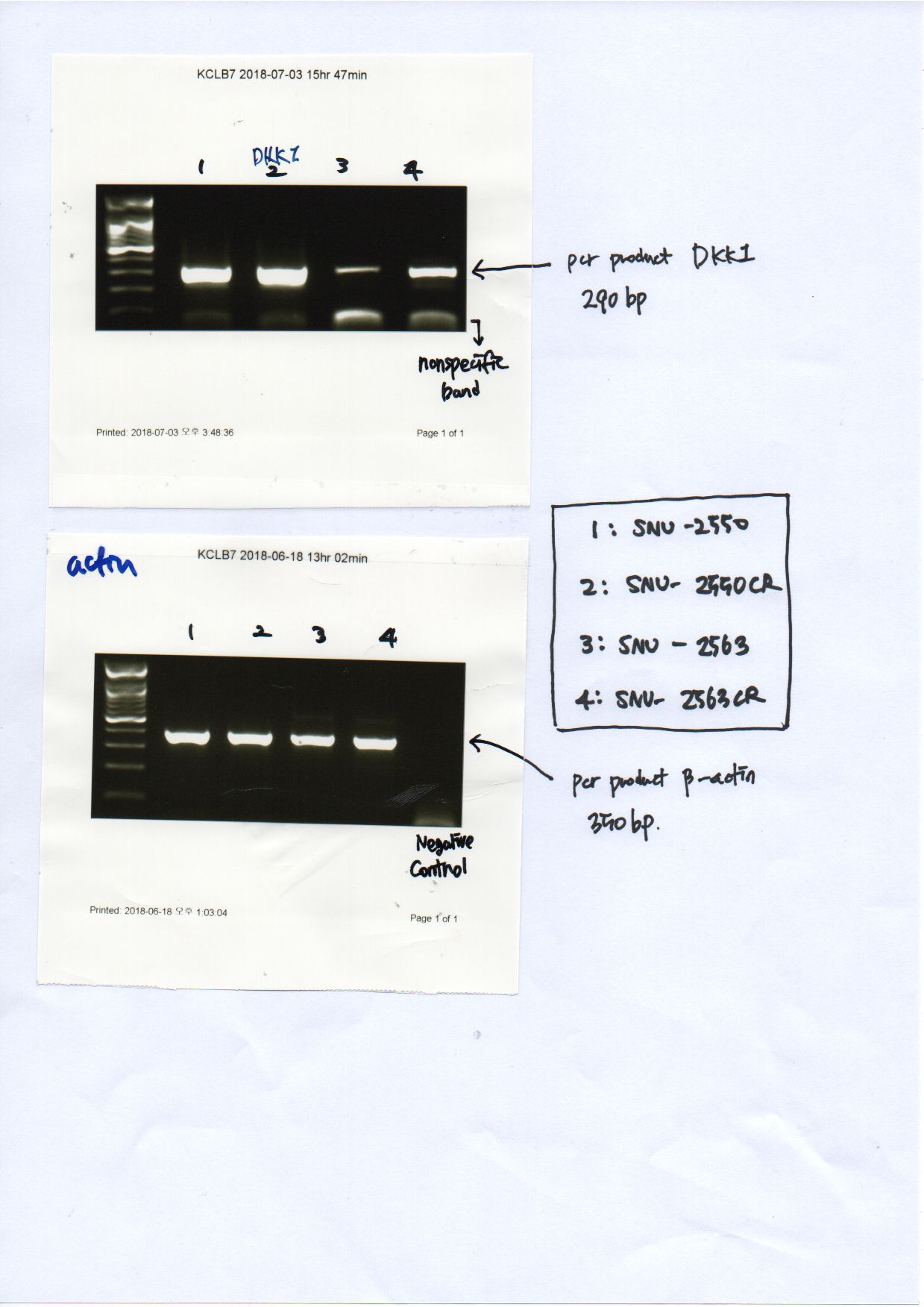
**

Raw and unprocessed image of Figure 4C (PCR loading gels film)


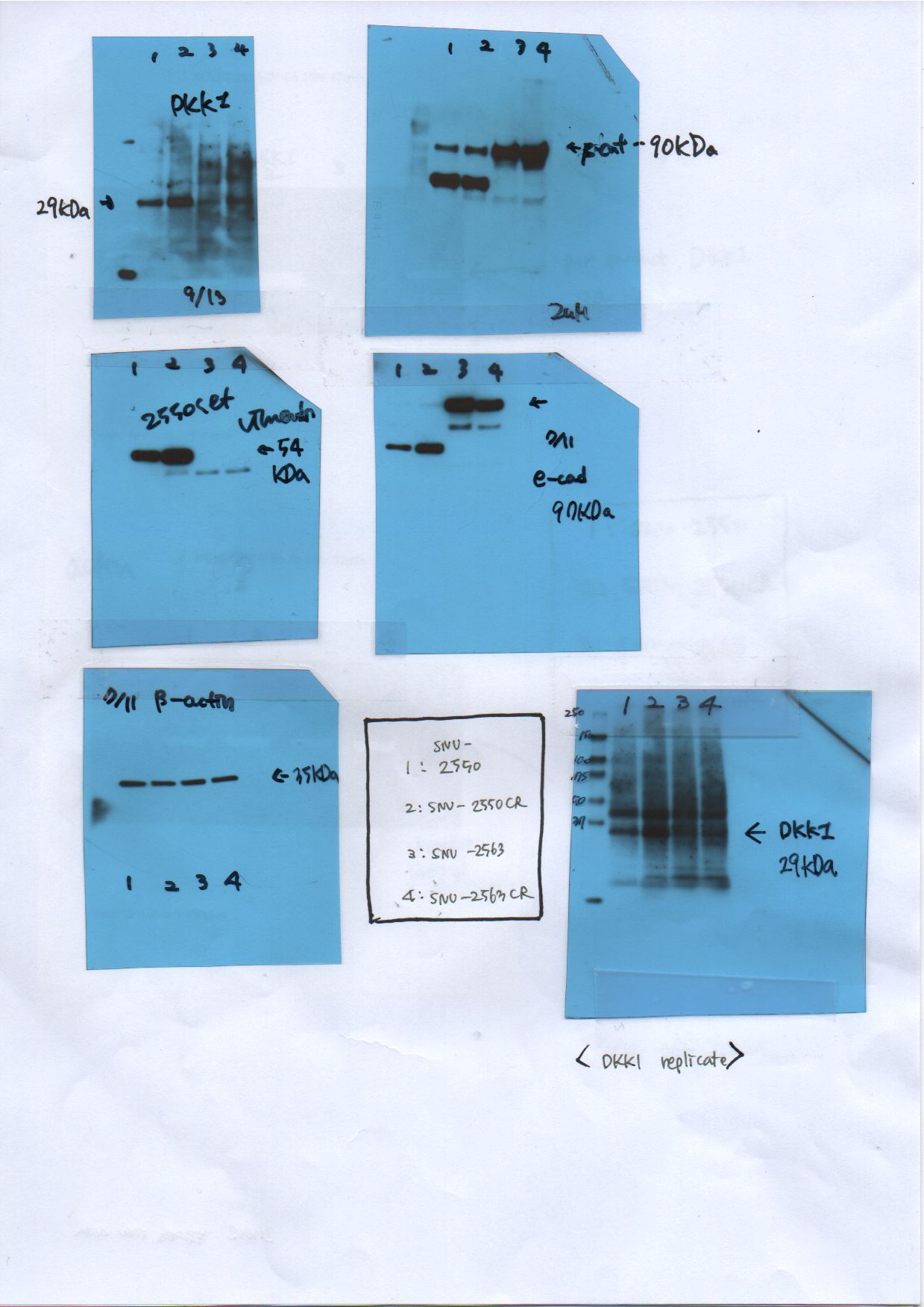


Raw and unprocessed image of Figure 4D (Western blot film)


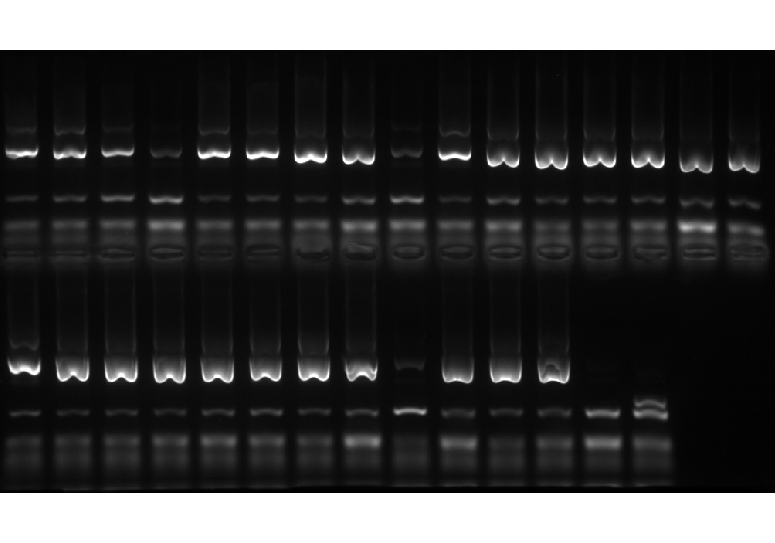


Raw and unprocessed image of Supplementary Figure 1 (Digital Image from Molecular Imager Gel Doc XR+ Imaging System)


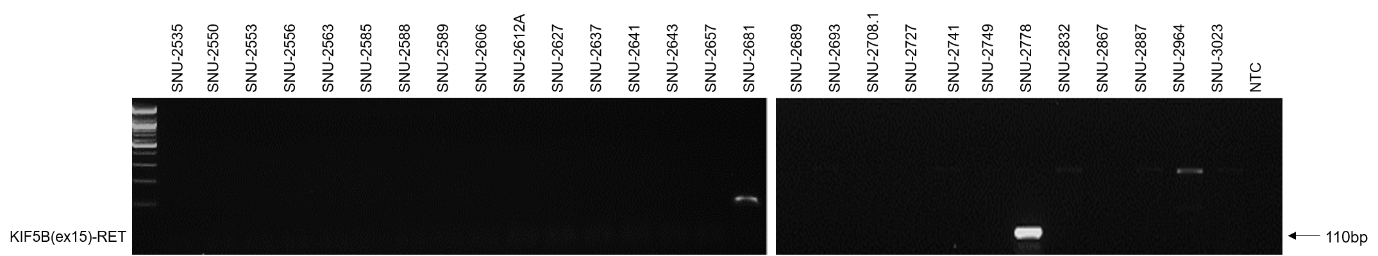


Raw and unprocessed image of KIF5B(ex15)-RET fusion gene from Supplementary Figure 4 (Digital Image from Molecular Imager Gel Doc XR+ Imaging System)


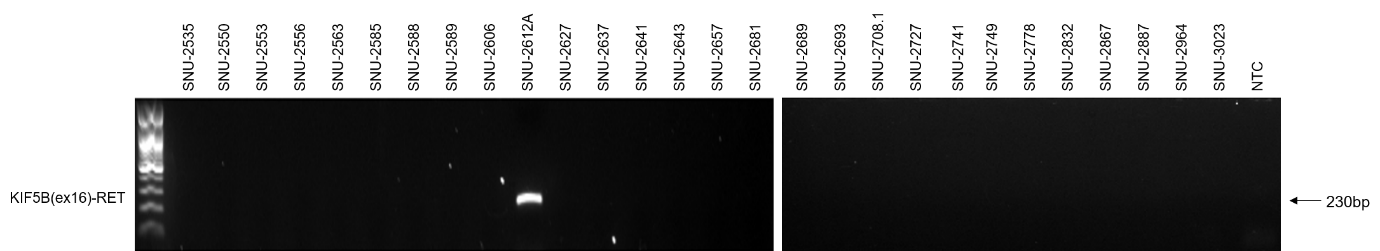


Raw and unprocessed image of KIF5B(ex16)-RET fusion gene from Supplementary Figure 4 (Digital Image from Molecular Imager Gel Doc XR+ Imaging System)


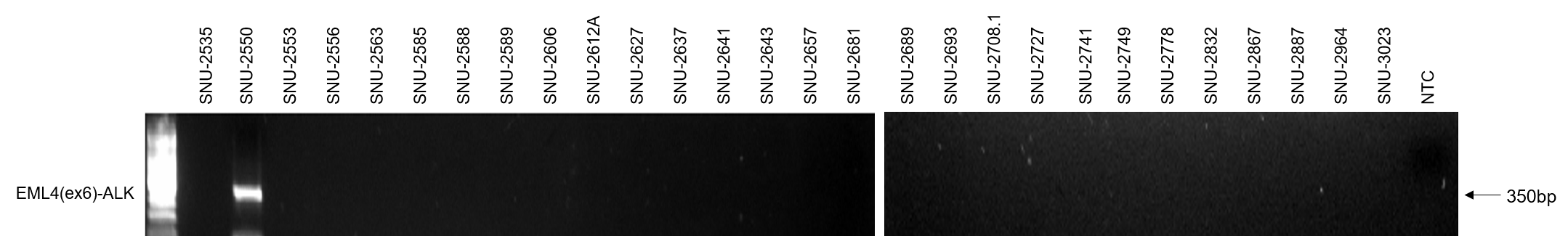


Raw and unprocessed image of EML4(ex6)-ALK fusion gene from Supplementary Figure 4 (Digital Image from Molecular Imager Gel Doc XR+ Imaging System)


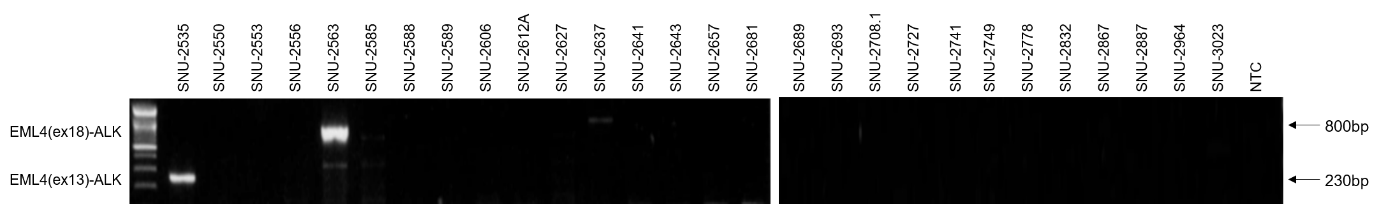


Raw and unprocessed image of EML4(ex13)-ALK and EML4(ex18)-ALK fusion gene from Supplementary Figure 4 (Digital Image from Molecular Imager Gel Doc XR+ Imaging System)


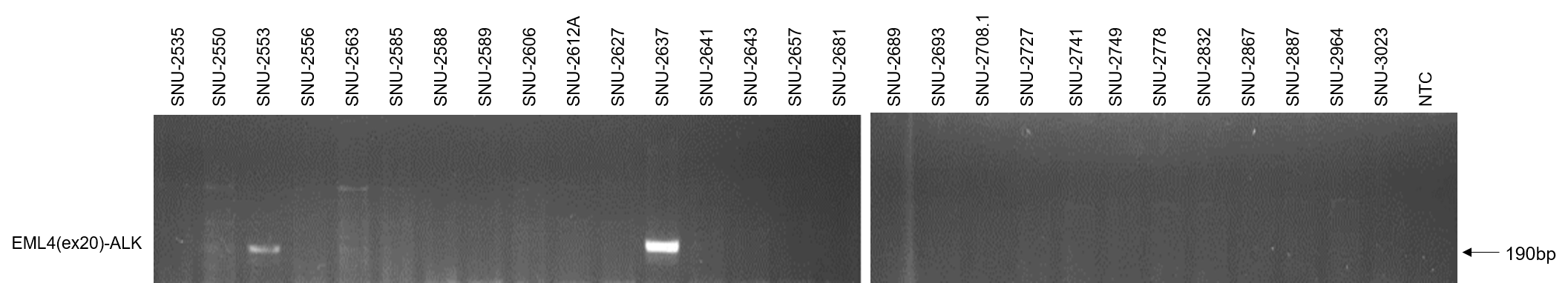


Raw and unprocessed image of EML4(ex20)-ALK fusion gene from Supplementary Figure 4 (Digital Image from Molecular Imager Gel Doc XR+ Imaging System)


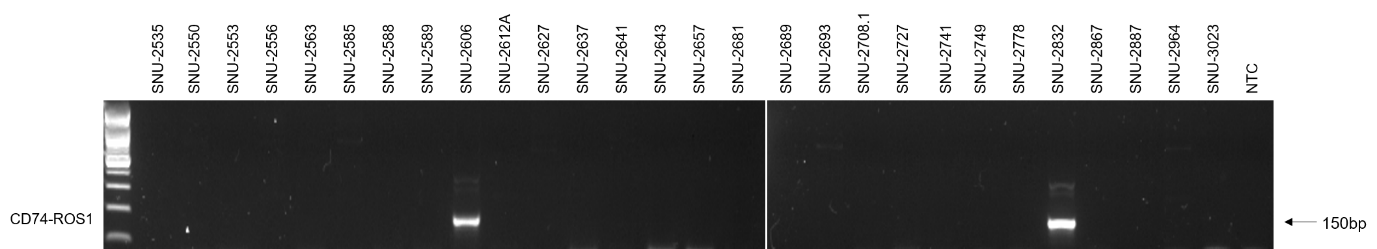


Raw and unprocessed image of CD74-ROS1 fusion gene from Supplementary Figure 4 (Digital Image from Molecular Imager Gel Doc XR+ Imaging System)


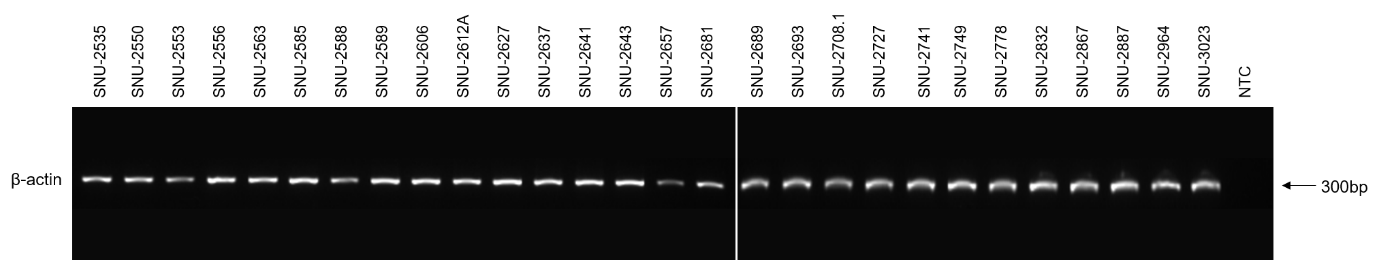


Raw and unprocessed image of β-actin gene from Supplementary Figure 4 (Digital Image from Molecular Imager Gel Doc XR+ Imaging System)


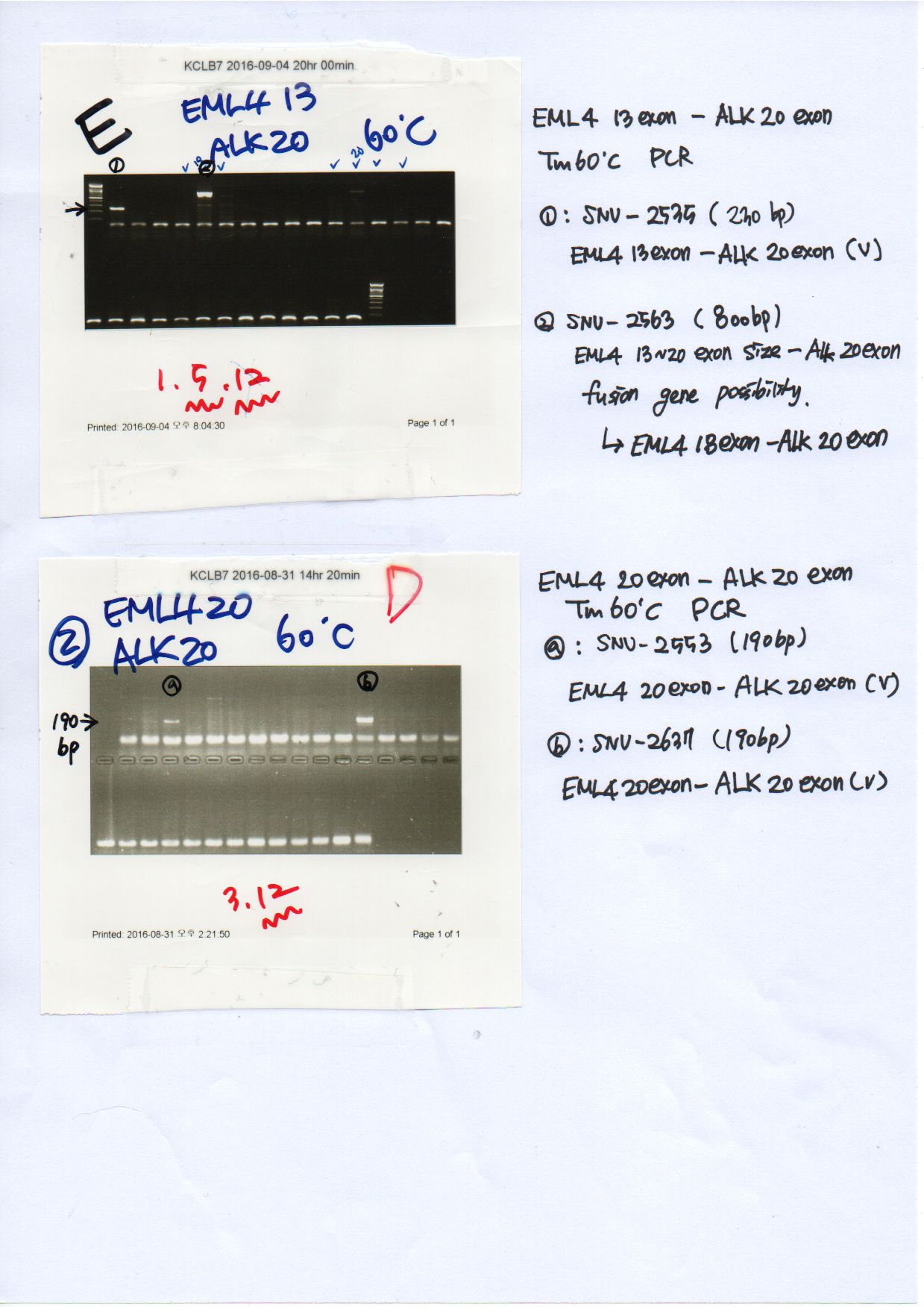


Raw and unprocessed image of EML4(ex13)-ALK fusion gene and EML4(ex20)-ALK fusion gene from Supplementary Figure 4 (PCR loading gels film)


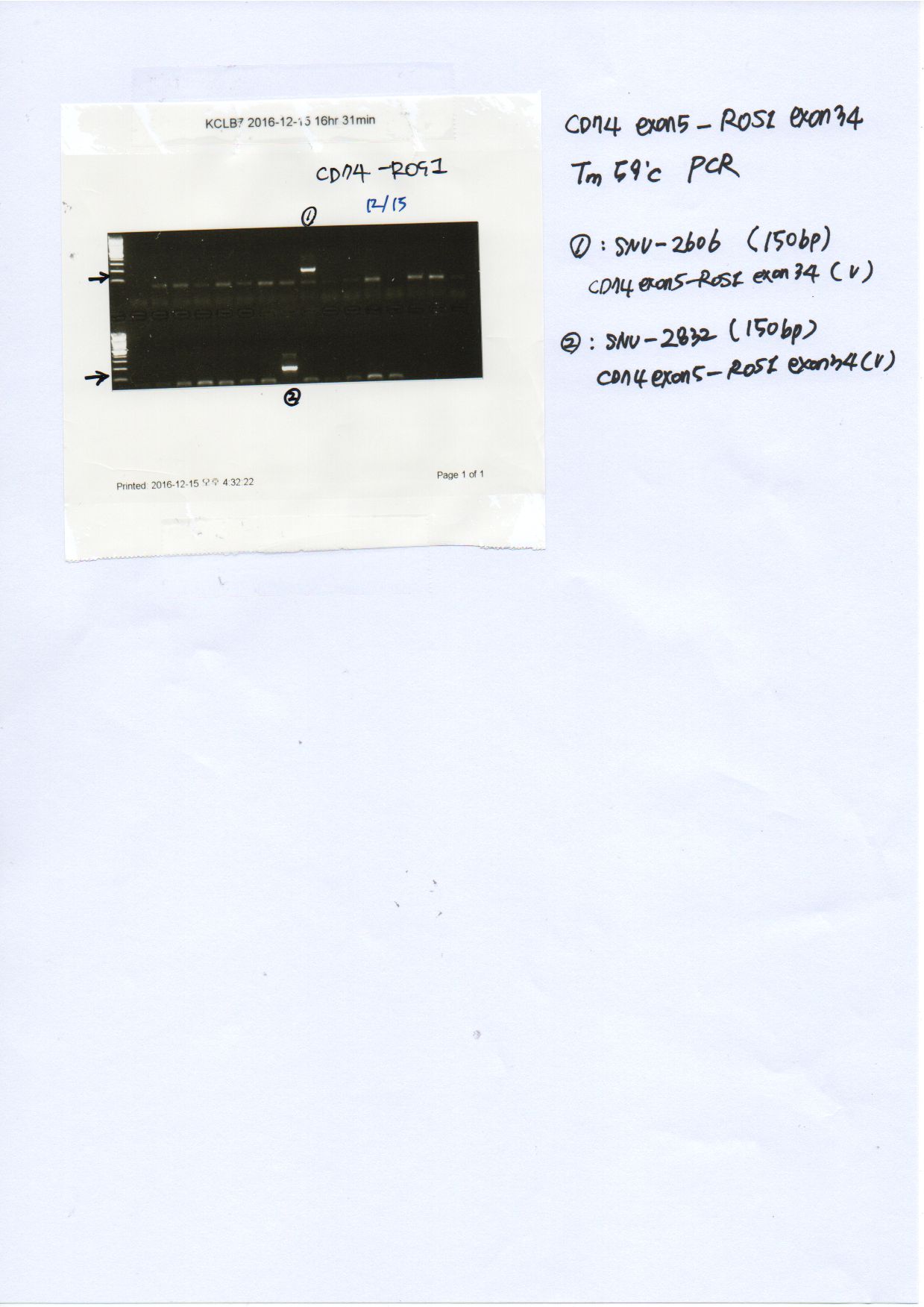


Raw and unprocessed image of CD74-ROS1 fusion gene from Supplementary Figure 4 (PCR loading gels film)


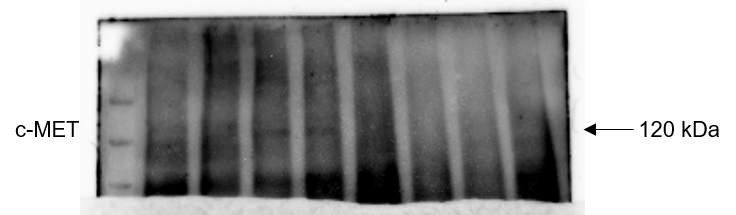


Raw and unprocessed image of MET protein from Supplementary Figure 6 (Digital Image from ChemoDoc Touch Imaging System)


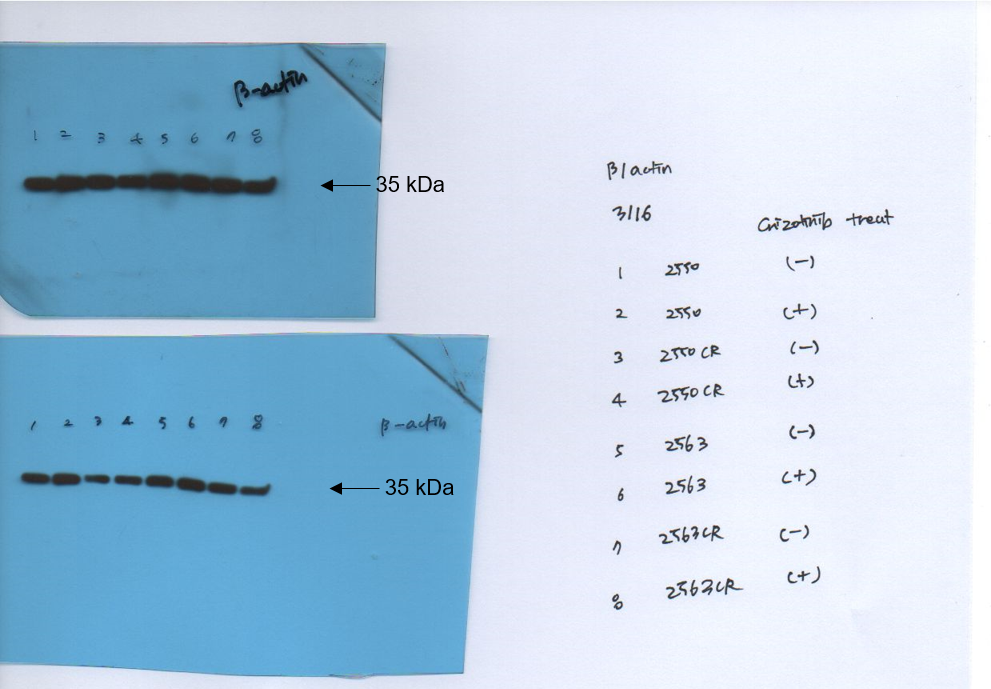


Raw and unprocessed image of beta-actin from Supplementary Figure 6 (Western blot film).
